# Determinism and stochasticity drive microbial community assembly and microbial interactions in calcareous glacier forefields

**DOI:** 10.1128/aem.00302-25

**Published:** 2025-05-15

**Authors:** Edoardo Mandolini, Maraike Probst, Anusha Telagathoti, Beat Frey, Luis M. Rodriguez-R., Flavio Fornasier, Nadine Praeg, Paul Illmer, Ursula Peintner

**Affiliations:** 1 Department of Microbiology, Universität Innsbruck 685630, Innsbruck, Austria; 2 Forest Soils and Biogeochemistry, Swiss Federal Research Institute WSL, Birmensdorf, Switzerland; 3 Digital Science Center (DiSC), Universität Innsbruck 27255, Innsbruck, Austria; 4 Research Center for Viticulture and Oenology (CREA-VE), Conegliano, Italy; University of Milano-Bicocca, Milan, Italy

**Keywords:** Alpine environment, calcareous bedrock, enzymatic activity, abundance-occurrence relation, microbial community assembly, neutral theory

## Abstract

Calcareous glacier forefields challenge prevailing ecological frameworks on microbial biodiversity and community assembly due to their unique bedrock. Early stages of soil development in these environments are notorious for their high turnover rates, demanding a high degree of replication for obtaining conclusive data. However, studies across different calcareous glaciers are still missing. Here, we robustly investigated both bacterial and fungal diversity, association networks, and assembly processes in four calcareous glacier forefields of the Alps, focusing on the earliest soil developmental stages (<25 years) early in the snow-free season. We found a diverse community of bacteria and fungi, potentially involved in P and N nutrient cycling. A core microbiome existing across all four locations suggests that certain microbes might be more successful colonizers of these ecosystems than others. Nearest taxon index revealed phylogenetically clustered microbial communities. These findings suggest that the distribution and colonization of some microbes were influenced by selective forces such as geography and climate during the early stages of soil development in calcareous glaciers. Interestingly, there were no common bacterial-fungal associations across the four locations, indicating that this habitat does not select for specific bacterial-fungal associations and that associations were driven by neutral processes. We discuss microbial communities and their interactions in these special calcareous glacier forefield habitats. Moreover, we present innovative approaches for studying microbial assembly that address both deterministic, intrinsic drivers, like specific microbial traits, and stochastic, extrinsic drivers, such as the opportunistic behavior of microbes.

**IMPORTANCE:**

Our study is based on three fundamental and unique approaches: (i) we utilize the early stages of soil development in four glacier forefields across the Alpine range. This design implies high replicability in a natural setting, which is crucial for drawing general conclusions. (ii) Our study investigates glacier forefields with calcareous bedrock directly after snowmelt. These habitats and periods remain surprisingly underexplored. (iii) Our results underline the relevance of bacterial-fungal associations in microbial community assembly alongside dispersal, drift, and natural selection. Taken together, our study provides new insights into the development of complex microbial communities, their stabilization and predictability, including ecological implications.

## INTRODUCTION

Microorganisms thriving in the soil and on rock surfaces of mountain environments play a crucial role in soil development and biogeochemical cycling. They affect plant establishment (1) and influence C-storage at the local scale (2) with unclear effects for the global C-budget. Microbes assemble into complex communities on any surface exposed by perturbations such as geomorphological instability, harsh climatic conditions, and glacial retreat. Recently deglaciated environments provide different ecosystem developmental stages along a well-defined chronosequence (1, 3, 4), where both deterministic and stochastic processes jointly contribute to the microbial community assembly across time and space at earliest stages of soil development (ESSD). These processes have been investigated in several other systems; however, the current retreating rate of Alpine glaciers in response to climate change (5) imposes pressure for describing these ecosystems, and their biodiversity, before they disappear (6, 7).

Pioneer microbial communities assemble following four fundamental ecological processes, namely dispersal, selection, drift, and diversification (8). In glacier forefields, spatial connection and distance are particularly influential for community assembly. Microbial colonizers are transported via both endogenous and exogenous sources (9, 10), and although considered passive (11), their dispersal probability varies across space, population size, species traits, and activity status. While bacteria mainly originate from subglacial and supraglacial sediments and glacial streams (9), fungal origins remain unclear. Nevertheless, the success of species establishment depends on the selective pressure acting in the recipient habitat.

Selection includes both abiotic conditions and biotic interactions. The soil microbial community structure in Alpine environments closely mirrors the complexity and high local-scale heterogeneity of these habitats. The list of environmental factors is extensive and includes climate, topography, geo-physicochemical characteristics of the parent material, and time (12). Naturally, historical contingencies (13) are virtually negligible at ESSD (1). Thus, the barren bedrock, climate, and incoming material are considered the major factors influencing microbial community assembly. Biotic interactions among microbes also affect the colonization success of species (14). While early colonizers can prevent or favor the establishment of new incomers by modifying their microenvironment, species may also affect each other by interaction. However, despite their significant roles in ecosystem development and function, microbial interactions remain difficult to measure. In glacier forefields, studies of association networks are increasing (15–20), but they mainly focused on a single taxonomic group (e.g., either plants, invertebrates, bacteria, or fungi) ignoring higher-order associations, such as among fungi and bacteria.

Community assembly is also influenced by ecological drift (8, 21). Ecological drift is tightly linked to the concept of functional redundancy, where different populations of a community overlap in their ecological function (22–24). Thus, functionally redundant populations are more susceptible to drift, where rare species may take over more abundant ones. This process is particularly important in glacier forefields, where environmental heterogeneity and dynamics (12) subject microbial communities to high compositional turnover (25), while still preserving the ecological function of the community (26).

Overall, patterns of microbial assembly processes in glacier forefields start to emerge. Bacterial assembly appears to be mainly driven by deterministic processes, while fungal assembly appears to be driven by stochastic ones (15, 17, 18). However, these observations were based on chronosequences that span decades or even centuries (1, 3, 4) and fail to capture the microbial dynamics within ESSD, where microbial assembly processes (e.g., biotic interactions) are expected to differ from older stages (1, 3, 16, 27). Indeed, in ESSD, bacterial and fungal communities are distinct from the rest of the chronosequence (18, 28), with higher complexity (16), functional diversity (26), and turnover rates than in older ones (29, 30). Given this dynamic nature, a comprehensive investigation of the assembly processes structuring microbial communities requires a high degree of replication. In this regard, studies researching general patterns in ESSD are still missing.

While past research has studied the microbial communities in glacier forefields worldwide (3), the microbial diversity and dynamics in glaciers with calcareous subglacial bedrock were rarely investigated (17, 20, 28, 31, 32). These environments are drastically different compared to other glacier forefields, for example, with siliceous subglacial bedrock, and harbor specific microbial communities (31, 33). In fact, calcareous glacier forefields present high levels of calcite (CaCO_3_) or dolomite [CaMg(Co_3_)_2_], resulting in alkaline soil pH and high cation exchange capacity, and thus ion retention or precipitation (34). It is also crucial to acknowledge the remarkable difference in hydrology between siliceous and calcareous bedrock. Soluble carbonate rocks such as limestone and dolomite result in a karst topography characterized by sinkholes and fissures (35), which act as rapid water drainage systems. The biodiversity, microbial interactions, and community assembly in calcareous bedrock environments are still largely unexplored and may challenge the existing knowledge of early ecosystem dynamics.

In this study, we investigated bacterial and fungal communities in four calcareous glacier forefields at ESSD (<25 years of ice retreat). We sampled all glacier forefields in the same seasonal period, i.e., directly after snowmelt. Usually, studies on glacier forefield microbial communities focus on summer conditions, when sites are easiest to access. However, this procedure ignores seasonal variations, which are common and strong in glacier forefields. As this approach limits our understanding of the ecosystem, we sampled immediately after snowmelt, thereby providing the first rare insights of glacier forefield microbial communities at colder temperatures compared to summer. This design adds seasonal information to our current knowledge and widens our understanding of the ecosystem. We aimed at (i) characterizing the microbial diversity across four calcareous glacier forefields, hypothesizing that a conserved microbiome exists across glaciers (i.e., core microbiome); (ii) predicting bacterial and fungal associations, hypothesizing that fungi and bacteria form complex networks at ESSD, but with some degree of commonality in associations/clusters that may be selected for regardless of location; and (iii) elucidating and comparing the drivers of bacterial and fungal community assemblies, hypothesizing that community assembly depends on a combination of factors, such as local physiochemical conditions, microbial traits, and microbial associations.

## MATERIALS AND METHODS

### Study locations and sampling procedure

Detailed information for all methods is provided in Supplementary Material and Methods. We sampled soils in the forefields of four glaciers of the European Alps (Table 1; Fig. S1): Hallstätter in Dachstein (D), Marmolada (M), Griessen (G), and Tsanfleuron (T). These forefields have sediments derived from calcareous bedrock, and their locations are well distributed across the Alpine range. All four proglacial areas are characterized by an Alpine climate typical of high elevations in the European Alps, with long winters and short summers. Like other Alpine glaciers, the ice front of these four glaciers has been constantly retreating since the end of the Little Ice Age, around 1850, rapidly exposing extensive deglaciated terrain (Table 1).

**TABLE 1:** General geo-climatical characteristics of the four investigated calcareous glaciers

		Dachstein	Griessen	Marmolada	Tsanfleuron
Location		Northern Limestone Alps, Austria	Helvetic nappes, Switzerland	Southern Limestone Alps, Italy	Helvetic nappes, Switzerland
Coordinates		47.48765, 13.618747	46.8422, 8.490461	46.441896, 11.859656	46.32335, 7.240629
Altitude		2,700–2,250 m	2,800–2,510 m	3,300–2,700 m	2,960–2,600 m
Exposure		NE (45°)	W (270°)	NE (45°)	E (90°)
Total ice front retreat		∼2,560 m	∼721 m	∼1,263 m	∼2,036 m
Parent rock material		Mesozoic limestones and dolomites	Mesozoic limestones, shales, and marls	Mesozoic latemar limestone and dolomite	Mesozoic limestones, shales, and marls
Soil type in the forefields		Rendzina–Lithic Leptosols	Rendzina–Lithic Leptosols	Rendzina–Lithic Leptosols	Rendzina–Lithic Leptosols
Air temperature (°C)^*a*^	Winter	−3.5^*b*^	−7.9^*c*^	−2.3^*d*^	−6.2^*e*^
	Summer	6.5^*b*^	1.1^*c*^	8.8^*d*^	2.4^*e*^
Rainfall (mm)^*a*^	Winter	89.9^*b*^	111.1^*c*^	146.5^*d*^	64.7^*e*^
	Summer	202.4^*b*^	201.2^*c*^	146.1^*d*^	148.7^*e*^
Plant species		*Arabis alpina* *Arabis pumila* *Cerastium uniflorum* *Poa minor* *Silene pusilla* *Saxifraga aphylla*	*Arabis bellidifolia* *Arabis caerulea* *Poa minor* *Saxifraga aizoides* *Saxifraga oppositifolia*	*Arabis alpina* *Arabis caerulea* *Cerastium uniflorum* *Poa minor*	*Bryum* sp. *Campanula cochleariifolia* *Cirsium spinosissimum* *Cerastium uniflorum* *Gentianopsis ciliata* *Hornungia alpina* *Linaria alpina* *Saxifraga aizoides* *Sedum atratum*
Monitoring program website		https://dachsteingletscher.info/	https://www.glamos.ch/en/#/B22-01 https://glamos.ch/en/mapviewer#/A51h-02	https://www.meteotrentino.it/index.html#!/content?menuItemDesktop=56	https://www.glamos.ch/en/#/B22-01

^*a*^ Monthly average of meteorological data from 01/10/2020 to 30/09/2021 across summer (May to September) and winter (October to April).

^*b*^ Values recorded at the closest weather station on Simonyhuette, ca. 1.5 km from our site and at 2,205 m.

^*c*^ Values recorded at the closest weather station Titlisboden (TIT2), ca 8.5 km from our site and at 2,149 m.

^*d*^ Values recorded at the closest weather station Pian Fedaia (Diga), ca 2.3 km from our site and at 2,063 m.

^*e*^ Values recorded at the closest weather station Tsanfleuron (DIA2), ca 1.5 km from our site and at 2,569 m.

Due to different fronts, sun exposures, and elevations, the four locations differ in calendar month of snowfall and snowmelt. In ESSD, seasonality plays an important role in determining the stability and shift of microbial communities in the soil. Consequently, the timing of soil sampling was carefully planned and carried out at each location as early in the snow-free season as possible in 2021, that is, in mid-July for D, end of July for T and G, and mid-August for M. At these times, the snow cover was just molten; areas still covered in snow, or with melting snow, were avoided. None of the soil samples collected were frozen at the time of sampling. For safety and representability purposes, we chose the day of sampling by stable, dry weather conditions on sampling day and 3–5 days before that.

At each location (D, G, M, T), we selected 10 plots (1 through 10), constituting safe sites, across the area proximal to the glacier terminus that has been deglaciated for a maximum of 25 years (Fig. S2; Table S1). The deglaciation dates were determined by combining publicly available resources (Table 1) and expert advice from the local institutions monitoring these locations every year. Recently deglaciated soils are poorly developed in these locations, mainly composed of barren sandy rock and loamy sand (36). Plants were absent on the barren ground proximal to the glacier terminus while pioneer vegetation gradually developed on the youngest terrains. Here, rock surfaces and gravels were patchily colonized by crustose lichens. Sporadic plants (one to two small individuals across 2 m^2^) colonized the small safe-site islands (Table 1).

At each sampling plot, five soil samples (A through E) were collected within a 1 m radius from the top 5 cm of the surface to account for local soil heterogeneity. Approximately 50 g of soil was sieved through a 2 mm sieve into individual Whirl-Pack bags and immediately placed on ice. Pebbles larger than 2 cm and plant materials such as roots or debris were avoided or removed. Overall, a total of 200 soil samples (50 samples per glacier) were gathered across all four glacier forefields.

Soil samples were kept on ice for the entire duration of the transport to the laboratory. Prior to storage at −20°C, soil was subsampled for DNA extraction and subsequent sequencing and geochemical and enzymatic analysis.

### Soil geochemistry and enzymatic activities

One soil sample from each plot was used for the soil physicochemical analysis (*n* = 40). All analyses were performed in technical duplicates. Their arithmetic mean was used for further analysis. Soil pH was measured as previously described (37). Plant-available P (µg g^−1^ dry weight [dw]), total P (TP; µg g^−1^ dw), and inorganic P (µg g^−1^ dw) were determined using the colorimetric molybdenum blue method (38). Prior to soil organic matter (SOM; %), water holding capacity (WHC; %), total carbon (TC; µg g^−1^ dw), total dissolved nitrogen (TDN; µg g^−1^ dw), dissolved organic carbon (non-purgeable organic carbon, DOC; µg g^−1^ dw), and elemental concentrations (µg g^−1^), soil samples were dried at 105°C overnight. Dry weight was determined by loss of weight.

Enzyme activities were quantified in soil extracts from every subsample (*n* = 200) as previously described (39) (Supplementary Material and Methods). Briefly, enzymes were extracted by heteromolecular exchange. Then, 20 µL of extracts was added to 50 µL of appropriate buffer (File S4). After the addition of specific substrate (2 g soil), enzyme activity was measured using a Synergy HT microplate reader (BIO-TEK). Quantitation of microbial biomass was carried out as previously described (40). Microbial biomass was expressed as micrograms of dsDNA.

### Community profiling of bacteria and fungi

Total genomic DNA was extracted from 2 g of each of the 200 subsamples using the E.Z.N.A. Soil DNA Kit. Communities were profiled targeting the V4 region of the prokaryotic (bacterial and archaeal) 16S rRNA gene and the ITS2 region of the eukaryotic (fungal and some groups of protists and green algae) ribosomal operon using Illumina MiSeq (v.2, 2 × 250 bp) (Microsynth AG, Balgach, Switzerland). Amplicon sequence variants (ASVs) for bacterial and fungal sequences were inferred using DADA2 (41). To this end, ASVs were clustered into operational taxonomic units (OTUs) with 97% similarity (Supplementary Material and Methods). Prokaryotic and eukaryotic OTUs were taxonomically assigned using the Silva Project’s v.132 (42) and UNITE Fungal ITS training set 10-05-2021 (43), respectively. Prokaryotic sequences identified as originating from organelles (chloroplast, mitochondria), as well as unclassified eukaryotic sequences, were removed prior to data analysis. Samples with low sequencing depth (<5,000 reads; G1C, G2E, G7E, M1A, M10E, T3E, T10C, T8A) were removed from both data sets, thus resulting in a total of 192 samples (D = 50; G = 47; M = 48; T = 47).

### Statistical analysis

The effect of locations on environmental variables, α-diversity metrics, and enzymatic activities was determined by analysis of variance followed by Tukey’s honest significant difference (HSD) tests. The nested design of samples within plots was considered. Following the ongoing debate on how to reliably assess α-diversity, we applied different methods for comparing α-diversity (rarefaction, subsampling, iterative subsampling). All methods tested gave similar results. As this manuscript does not focus on α-diversity assessment, we report the classical method. Relationships between environmental variables and locations were visualized using principal component analysis (PCA), while relationships between microbial community compositions and locations were visualized by non-metric multidimensional scaling (NMDS) based on Bray-Curtis dissimilarities of OTU count table. The significance of clustering was evaluated by permutational multivariate analysis of variance (PERMANOVA), considering the nested design of samples within plots. Variation partitioning was conducted to evaluate the effects of geography and environmental variables on the bacterial and fungal communities (Supplementary Materials and Methods). The correlations between enzymatic activities and soil properties were calculated; their relationship to microbial communities was evaluated by PERMANOVA. Then, enzymatic activities were normalized across enzymes to range from 0 to 1 prior to visualization with a heatmap.

To determine a “core” microbial community (Venn diagrams), we only included bacterial OTUs that occurred in ≥10 samples (out of 50; 20% occupancy) and fungal OTUs that occurred in ≥5 samples (out of 50; 10% occupancy) in each glacier forefield. These thresholds were chosen based on data distribution, i.e., after a drop in counts and along a stable plateau, where the choice of threshold would not change the community composition (Fig. S6). This filtering did not remove abundant OTUs. Because barren soil may be inoculated by microorganisms residing in the snow after snowmelt, we cross-checked bacterial and fungal genera diversity (richness, taxonomy, and relative abundance) typically reported from snowpacks in the Alps (9, 44, 45) with our core microbiome data set to exclude possible bias in our soil microbial composition. Abundance-occupancy relationships of bacterial and fungal OTUs were obtained by dividing the occupancy of each OTU within a forefield by its mean relative abundance across the samples of that glacier. Spearman’s rank correlation (rho) was computed to test the correlation between site occupancy and mean relative abundance.

Individual association networks were inferred for each forefield based on bacterial and fungal OTU tables using SPIEC-EASI (46). In order to reduce network complexity, we only included bacterial and fungal OTUs that occurred in at least 10 and 5 samples, respectively, in each glacier forefield (Fig. S6), as described for the core microbiome. In order to test if the frequencies of observed fungal-fungal, bacterial-bacterial, and bacterial-fungal associations among glacier networks deviate from the frequencies expected by chance, we randomly generated 999 random networks for each network inferred (Supplementary Material and Methods). Bacterial-fungal associations were extracted from the resulting networks (i.e., fungi-fungi and bacteria-bacteria associations were removed). “Core” microbial associations, i.e., pairwise associations present across glaciers, were inspected with a heatmap and by comparing clusters of nodes (modules) among networks.

The relative influence of stochastic or deterministic processes on the microbial community assembly was calculated based on mean nearest taxon distance and the nearest taxon index (NTI) (47, 48). The estimated contribution of assembly processes was based on the Sloan neutral community model (NCM) (49) and was conducted by predicting the relationship between the occurrence frequency of species in a set of local communities (i.e., individual subsamples) and their relative abundance across the global metacommunity (i.e., all four glaciers) (50). Species with an occurrence frequency higher than the neutral distribution are considered to persist (selected-for), whereas species with an occurrence frequency lower than the neutral distribution are considered likely lost from the ecosystem (selected-against). OTUs falling within the neutral distribution are likely driven by stochastic processes (neutral OTUs) (Figure 5A). To assess the proportion of neutrally and non-neutrally distributed OTUs within the inferred networks, the OTU contributions predicted by the model were fit to the corresponding network nodes. All analyses were run in R Statistical Software (v.4.0.3) (51).

## RESULTS

### Environmental variables and soil properties

**Fig 1:**
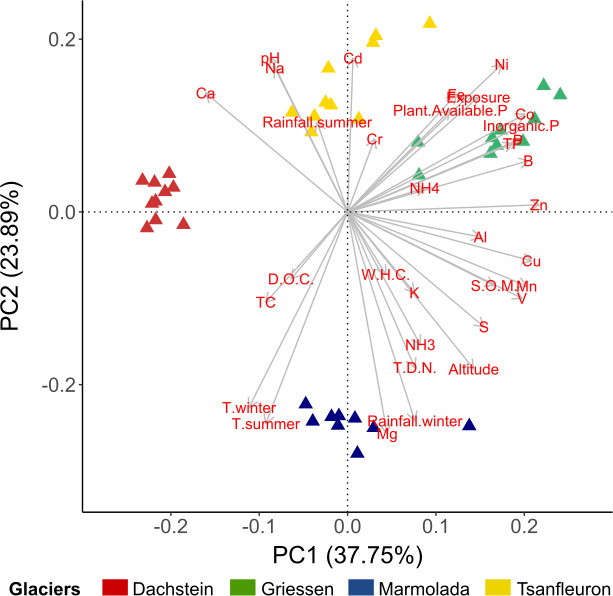
PCA of the four glacier forefields with respect to the environmental variables analyzed. Triangles indicate the soil properties for the plots of each location. Arrows indicate the contribution of each variable. Abbreviations: NO_3_^-^, nitrate; NH4+, ammonium; P, phosphorous; PO_4_^3-^, phosphate; TP, total phosphorus. Statistics: *R*^2^ = 0.6787, *F*_3,36_ = 25.35, *P* = 0.001.

Overall, the four different glacier forefields were heterogeneous, yet relatively distinct in their properties (Fig. 1). Despite heterogeneity and location effects, to some degree, the four glacier forefields were comparable based on the following criteria: WHC and the content of DOC, NH_4_^+^, and K (Table S2; Fig. S3). G and T had the highest contents of plant-available P, inorganic P, TP, elemental P, B, Co, Fe, and Ni. D and M had the highest content of TC. With regard to soil mineralogy, there was some variability across the four different glaciers. Across all locations, T had the highest contents of Cd, Cr, Fe, and Na; D had the lowest content of SOM, plant-available P, inorganic P, TP, elemental P, Al, B, Co, Cu, Fe, Mg, Mn, Ni, S, V, and Zn; G and M soil minerology values were intermediate between T and D (Table S2; Fig. S3).

### Enzymatic activity

**Fig 2:**
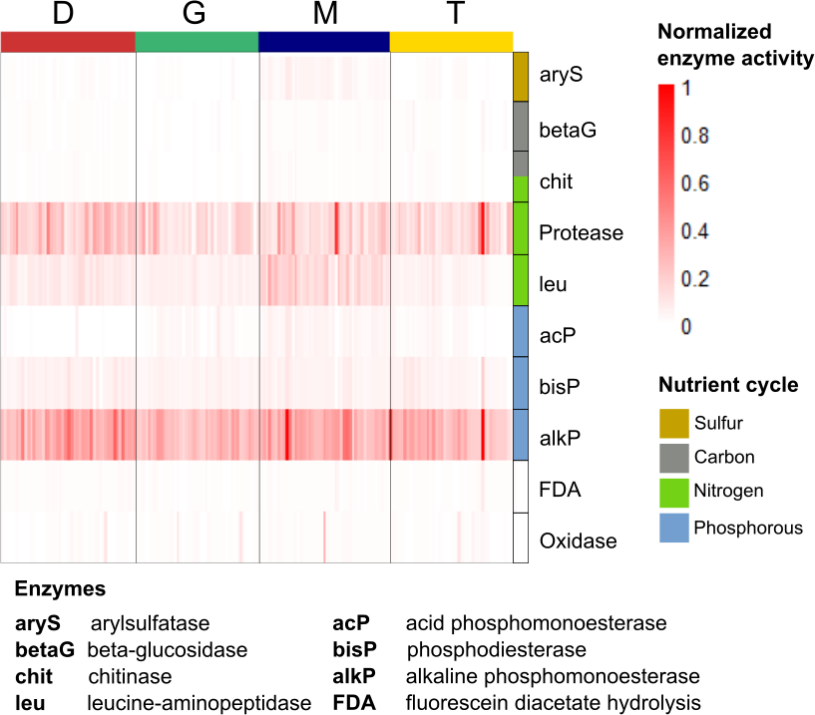
Enzyme activities measured in the four glacier forefield soils. Values were normalized across all enzymes between 0 and 1 for readability.

Our analyses showed that the enzymatic activities measured were related to N and P biogeochemical cycles in the glacier forefield soils (Fig. 2). Out of the 10 enzyme activities measured, alkaline phosphomonoesterase (alkP) was highest in absolute activity (30.0 ± 10.5 nmol 4-methylumbelliferone g^−1^ dry soil h^−1^) across all locations, followed by proteases (14.0 ± 3.4 fluorescence units) (Table S4). No activity was measured for the other enzymes (arylsulfatase [aryS], β-glucosidase [betaG], chitinases [chit], acid phosphomonoesterase [acP], phosphodiesterase [bisP], fluorescein diacetate hydrolysis [FDA], oxidase); slight activity for leucine-aminopeptidase was measured only at Marmolada (Fig. 2).

Total dissolved nitrogen (TDN) and Mg were positively correlated to the higher enzymatic activities in M. Mg correlated with aryS and leu, whereas TDN correlated with aryS, acP, and alkP. Correlating the microbial community composition to enzymatic activities resulted in significant relations only between leu and the bacterial community composition (13.5% variance, *P* = 0.001); other combinations were insignificant.

### α-Diversity and core microbial communities

The sequencing data set contained a total number of 8,272,364 bacterial/archaeal V4-16S rRNA and 5,768,076 fungal ITS2 gene reads. These reads were assigned to 26,290 bacterial, 165 archaeal, and 9,225 fungal ASVs. After OTU clustering, the final data set contained 13,452 bacterial, 95 archaeal, and 4,312 fungal OTUs in a total of 192 samples (File S1a and b). Sequencing depths did not differ among locations (Fig. S4). Rarefaction curves showed saturation for both bacteria/archaea and fungi (Fig. S5).

The bacterial communities were dominated by *Pseudomonadota* (37%), followed by *Actinomycetota* (14%), *Acidobacteriota* (12%), *Bacteroidota* (8%), *Chloroflexota* (7%), *Planctomycetota* (4%), *Cyanobacteriota* (2.5%), *Verrucomicrobiota* (2.5%), and *Gemmatimonadota* (2%). Combined, the remaining 31 bacterial phyla represented 4% of the total bacterial sequences. The fungal communities were dominated by *Ascomycota* (52%), *Basidiomycota* (35%), *Chytridiomycota* (10%), and *Mortierellomycota* (1%). Combined, the remaining 13 fungal phyla represented 2% of the total fungal sequences only. The archaeal community was extremely low in abundance (<1%) and sporadic, thus not further explored. Notably, the only exception was a specific OTU (OTU1209) assigned to the Nitrososphaeria class within Thaumarchaeota (File S1a), which was only found in G and T with an abundance of 4.5% and 2% of the total 16S rRNA gene data sets, respectively.

**Fig 3:**
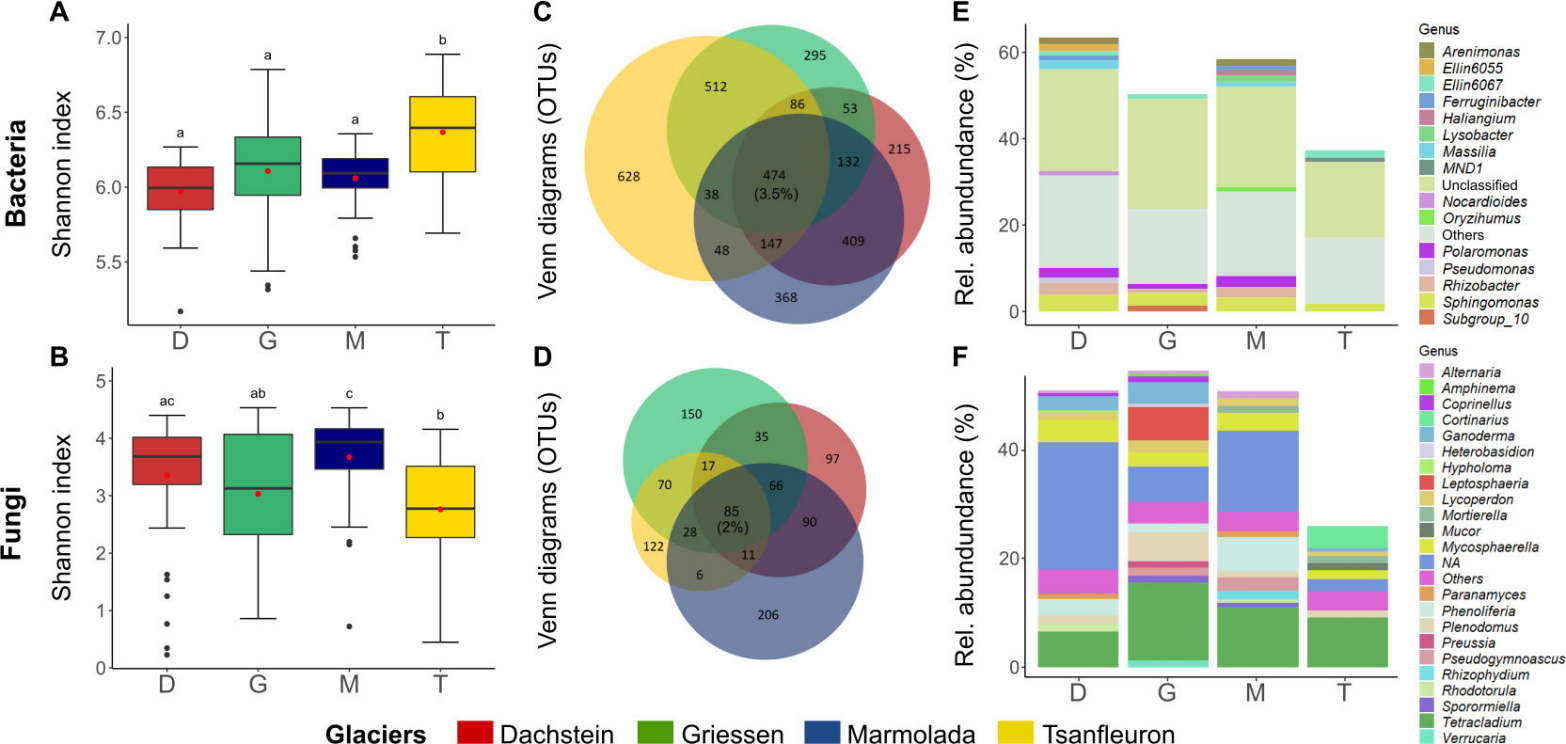
α-Diversity across glacier forefields. (A and B) Shannon index of microbial communities detected at the different locations. Horizontal lines represent the median, while the boxes represent the inter-quartile range of the first and third quartiles. The vertical lines (whiskers) represent the maximal and minimal values. Points within each boxplot represent the means. Letters indicate differences between glaciers in bacterial (*F*_3,188_ = 19.77, *P* < 0.001) and fungal (*F*_3,188_ = 8.32, *P* < 0.001) diversity. (C and D) Venn diagrams including the number of bacterial OTUs that occurred in at least 10 samples (out of 50) in each location and fungal OTUs that occurred in at least five samples (out of 50) in each location. The number and percentage in the middle are the number of OTUs shared across all glaciers (core). (E and F) Relative abundances of bacterial and fungal genera belonging to the core of the Venn diagrams; relative abundances of unique bacterial and fungal genera are not shown; bacterial and fungal genera with relative abundances below 1% and 0.5%, respectively, were summarized in Others.

Bacterial and fungal α-diversity indices were more comparable among D, G, and M; T often differed from the other three. While the Shannon indices of D, G, and M were similar, in T, the bacterial and fungal Shannon index was highest and lowest, respectively (Fig. 3A and B). Also, the bacterial and fungal OTU richness was similar for D, G, and M (1,100 ± 210 bacterial and 210 ± 87 fungal OTUs); T was richer in bacterial and poorer in fungal OTUs than the other three locations (1,400 ± 370 bacterial OTUs; 130 ± 70 fungal OTUs). Similar trends were observed for Simpson index, Faith’s phylogenetic diversity (PD), and evenness (Table S3; Fig. S7).

We found a small core community composed of 474 bacterial (474/13,452, 3.5%) and 85 fungal (84/4,312, 2%) OTUs that were detected among all four glacier forefields (Fig. 3C and D; File S2). Importantly, the core OTUs accounted for a high proportion of the overall abundances (Fig. 3E and F), representing up to 60% and 40% of the total bacterial and fungal relative abundance in the soils, respectively. Besides classified taxa, the core also contained a high number and large proportion of unclassified (23% bacteria; 12% fungi, on average) and low-abundance OTUs. Bacterial and fungal genera typically reported as dominant in snowpacks of the Alps were mostly absent from the core microbiome we detected. The exceptions were the yeast genus *Rhodotorula* and bacteria of the widespread genus *Pseudomonas,* which were present in very low abundance.

Last but not least, we observed a significant abundance-occurrence relation (A-O relation) for all four locations and for both bacteria and fungi, that is, the most abundant OTUs were most distributed across samples (Fig. S8).

### Microbial networks

Networks were inferred separately for each location, as we wanted to minimize data set sparsity, thereby improving networks’ robustness. Using separate inference, we also benefit from comparing network statistics across locations, which allows for a better grasp on calcareous glacier forefields, in general, via similarities across locations.

Bacterial-fungal associations accounted for 30% of the total associations (bacteria-bacteria = 60%, fungi-fungi = 10%), and they were further investigated (File S3). For all glaciers, networks were very dense, and statistics were comparable (Table 2), thereby indicating that these network statistics are typical for calcareous glacier forefields in ESSD. Across networks, although there were much more bacterial than fungal nodes, fungi had the most edges: on average, bacterial nodes had 8.13 ± 1.4 edges, while fungal nodes had 33.1 ± 4.45 (Fig. S9). Among bacterial nodes with the highest number of edges (>20), there were no OTUs commonly found across locations. Highly connected fungal nodes (>100 edges) detected across all glaciers were annotated as *Verrucaria latebrosa*, *Betamyces americae-meridionalis, Sporomiella intermedia, Amphinema byssoides,* and *Plenodomus biglobosus*. While, across glacier forefields, the median number of edges of bacterial core OTUs was lower than for non-core bacterial OTUs, the median number of edges of fungal core OTUs was higher than for non-core fungal OTUs (Fig. S10).

**TABLE 2:** Network statistics for bacterial-fungal microbial networks of the four glacier forefields

	Dachstein	Griessen	Marmolada	Tsanfleuron
Linkage density (complexity)^*a*^	10.9 (max: 40)	13.7 (max: 49)	14.9 (max: 49)	11.4 (max: 65)
Edge density	0.56	0.65	0.68	0.5
Nodes (OTUs)	1,950	2,099	2,196	2,257
# Bacteria	1,527	1,613	1,645	1,920
# Fungi	423	486	551	337
Total number of edges (connections)	10,651	14,406	16,411	12,825
Modules	29	27	24	25

^*a*^ Average number of edges per node.

All OTUs belonging to the core microbiome were found in the four networks. However, there were no bacterial-fungal pairwise associations detected across all glacier forefields in our study. Only three pairwise associations were shared among D, G, and T (Fig. S11). This suggests that in our samples, there were no frequently occurring bacterial-fungal associations. This was coherent with the number of expected shared bacterial-fungal associations predicted by random network inference (*P* = 0.468).

Since we did not detect core associations across locations, we hypothesized to find comparable modules (OTU clusters) among them. However, based on fluvial plots and cluster dendrogram (Fig. S12), we could not find comparable modules across the four glacier forefields.

### Processes influencing the assembly of bacterial and fungal communities

**Fig 4:**
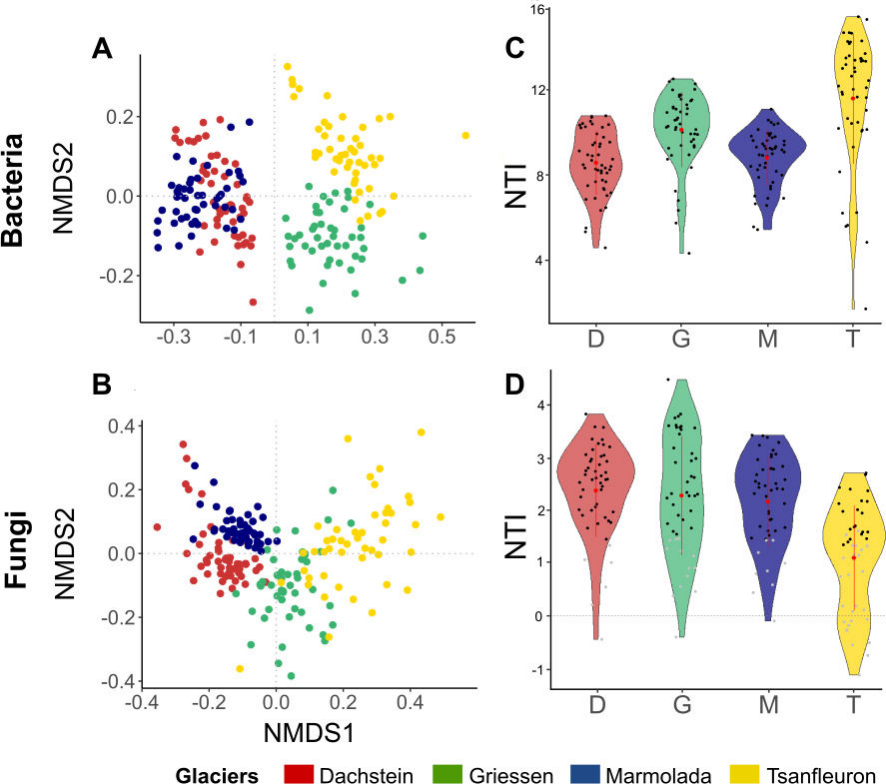
Community-level (β) diversity of bacterial and fungal communities among the glacier forefields. (A and B) NMDS of bacterial and fungal communities among locations (bacterial NMDS stress value = 0.114; PERMANOVA *R*^2^ = 0.35, *P* = 0.001; fungal NMDS stress value = 0.158%; PERMANOVA *R*^2^ = 0.19, *P* = 0.001). (C and D) NTI contributions of the bacterial and fungal communities for each glacier. Points represent NTI values for microbial communities within each sample plot, colored based on their difference to the expected value of zero (black, community significantly different, *P* < 0.05; gray, community not significantly different, *P* > 0.05; two-tailed *t*-test). Red points within each violin represent the means (*n* = NTI value scores), while the vertical lines represent the standard deviation. The kernel probability density of the data at different values is represented by the width of the violin graphs, that is, a wider part of the violin represents a higher density of scores at that particular level (i.e., for that location), while a narrower part of the violin represents a lower density of scores. Community (gray points) whose NTI is close to zero (horizontal dashed line) exhibits little phylogenetic clustering and indicates that the community is phylogenetically random.

Microbial communities differed among the four glacier forefields, and 66% and 43% of the variation within the bacterial and fungal composition, respectively, could be explained by location and nesting of plots within samples (Fig. 4A and B). Particularly, D and M communities were more comparable to each other and were separated from the communities detected at T and G. The relation between the microbial community composition and environmental variables was generally higher for the bacterial community than for the fungal one (Table S5). However, in both cases, the size effect of individual environmental variables was usually low (< 5%; Table S6); only inorganic P explained >10% variance within the bacterial community (Table S5).

Because the environmental variables alone explained very little variance within the microbial compositions, we used variance partitioning to investigate the relative role of environmental variables and spatial distances. Together, the environmental and spatial components explained a larger proportion of variance in the bacterial model (34.6%) than in the fungal one (16.8%). In both cases, most of the explained variance was attributed to the interaction between the two factors (bacteria = 31.6%; fungi = 10.0%). The contribution of environmental variables to variance explained was marginal (bacteria = 4.8%; fungi = 7.7%).

As an important proportion of compositional variance remained unexplained, we examined the relative influences of stochastic and deterministic processes on the microbial community assemblies by analysis of phylogenetic structure. All the mean values of NTI across all bacterial and fungal communities were significantly higher than the expected value of zero, meaning that microbial communities of both bacteria and fungi were more phylogenetically clustered than expected by chance in all glacier forefields (NTI >0, homogeneous selection) (Fig. 4C and D). This agrees with the Faith’s PD values found for both bacteria and fungi (Fig. S7). In all plots, bacterial communities were phylogenetically clustered (Fig. 4B). For the fungal communities, there were plots with low phylogenetic clustering, thereby suggesting a stronger influence of neutral processes in those soil plots (Fig. 4D, gray points).

**Fig 5:**
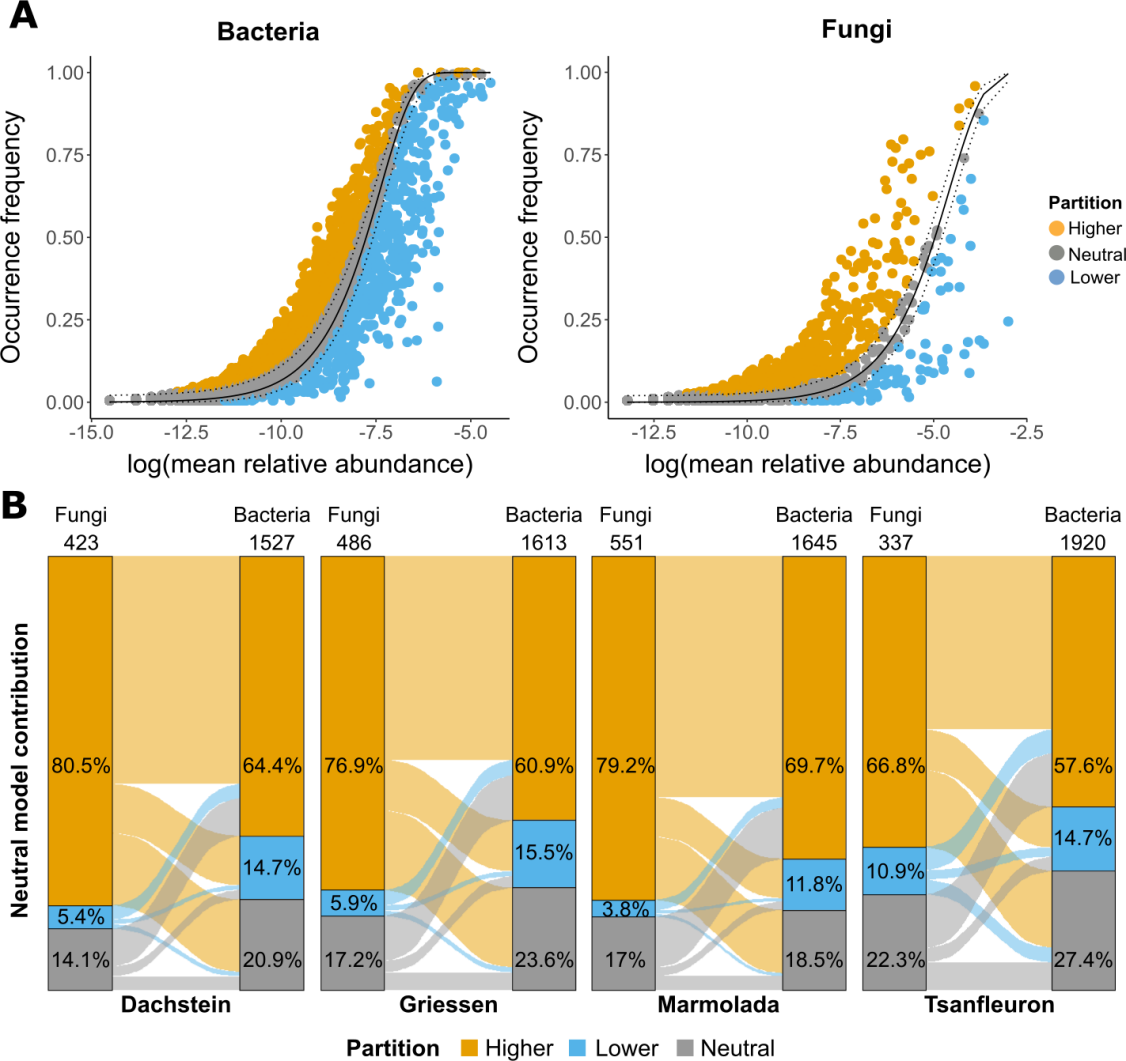
(**A**) Estimation of the neutral processes for bacterial and fungal communities of the four glacier forefields based on the Sloan NCM. The species (OTUs = points) that occur more and less frequently than predicted are shown in orange and light blue, respectively. Dashed lines represent 95% confidence intervals, and the species (OTUs = points) falling within the confidence intervals (gray) are considered neutrally distributed. (**B**) NCM of each OTU (node) in pairwise associations of the calculated networks. Within each glacier forefield, the *y*-axes are composed of fungal and bacterial nodes (number of nodes on top of each axis) and contain the percentages of nodes that were assigned to a specific partition (colored strata) by the NCM. Flow segments connect a fungal node to its bacterial node as pairwise association predicted by the network.

The NTI measure is based on phylogenetic distances (i.e., community-level). As the influence of the environmental variables on the microbial community composition was low, we tested if the homogeneous selection observed for the fungal and bacterial communities would hold. Therefore, we assumed no phylogenetic relations among OTUs (i.e., microbial distribution across communities), and we calculated NCM (49). This model estimates distributions expected by chance, thereby predicting which OTUs followed neutral distribution (random dispersal and drift) and which ones followed selection. Overall, the greatest proportion of OTUs in each glacier’s community was composed of OTUs whose distribution was as expected based on their abundances (neutral, on average, bacteria = 59%; fungi = 64%) (Fig. 5A; Table S6). Thirty-three percent of the OTUs were more widespread than expected by their abundances (higher = selected-for). Less than 6% of the whole community was composed of abundant OTUs that occurred less frequently than expected (lower = selected-against). Conversely, many of the OTUs found in the core microbiome were more widespread than expected (bacteria = 49%; fungi = 81%), suggesting that these OTUs are specifically adapted to, and selected by, the habitat (Table S6).

Next, we checked how neutral, selected-for, and selected-against OTUs were integrated into the networks (OTU = node). For each glacier, the greatest portion of pairwise associations was composed of nodes that were estimated to be selected-for (Fig. 5B); nodes estimated to be neutral or selected-against accounted for only small proportions. Thus, fungal nodes had a higher proportion of selected-for nodes than bacterial ones when considering their lower number in the networks. Moreover, the pattern of associations across these categories was comparable among locations. Finally, both bacterial and fungal key nodes were selected-for nodes (Fig. S13).

## DISCUSSION

This study investigated the diversity, association networks, and community assembly drivers of bacterial and fungal communities in the ESSD (<25 years ice-free soil) of four glacier forefields with calcareous parental material during the same seasonal timeframe.

Extracellular enzyme activities can effectively reflect the functions of microbial communities depending on the dynamic balance of metabolic requirements and nutrient availability (52). In the four calcareous glacier forefields addressed in this study, we found activity of enzymes associated with the P and N cycles in ESSD (Fig. 2). The deterministic effect of inorganic P on the community composition detected here fits well with this overall picture. Limited enzymatic activities are expected at this soil stage, where the primary nutrient sources are recalcitrant C and minimal amounts of allochthonous organic material (e.g., plant debris, insects) or necromass (26, 53, 54). High enzymatic activity related to the P cycle in the Puca glacier forefield in the Peruvian Andes indicated strong P limitation in calcareous bedrocks (32, 55), independent of snow cover, due to elevated ion retention (34). This is very likely also the case in the glacier investigated in our study. Other studies at the Puca and Hailuogou glacier forefields have shown high activity of N- and C-cycling enzymes during snow-free periods (20, 56–58), suggesting that nutrient limitation may vary seasonally. However, generalized patterns in nutrient limitation remain vague, and higher resolution temporal sampling is required to fill this knowledge gap.

Our results also reveal that calcareous glacier forefields support highly diverse microbial communities (Fig. 3A and B), which are somewhat comparable to those found in barren soils of other glacier forefields like Grisons (33), ESSD of Hailuogou (17), Rootmoos (28), and other non-calcareous forefield soils (16, 19). Considering the expected losses of bacterial and fungal diversity by OTU clustering and DNA extraction, the results of high α-diversity values point toward an even higher expected α-diversity in ESSD. This high diversity, combined with a substantial presence of unknown bacterial and fungal community members (28), provides opportunities for discovering new taxa (59). This is particularly crucial given that these ecosystems are disappearing due to global warming. In addition, the A-O relation (60, 61) revealed that the most abundant OTUs were widespread within and across each forefield (Fig. S8; Fig. 3C through F), while a long tail of rare, locally limited OTUs was also present in each location. These ecological patterns highlight the potential influence of rare species on microbial diversity. Our results highlight the uniqueness of the microbial communities in these habitats and are therefore a strong argument for their further exploration.

One aim of this study was to investigate whether there is a typical microbial community in calcareous glacier forefields. We found a core microbiome consisting of a low number of OTUs (Fig. 3C and D), which, however, accounted for a high proportion of the total bacterial (60%) and fungal (40%) reads (Fig. 3E and F). Among the most abundant bacterial core OTUs, we found taxa belonging to the genera *Polaromonas*, *Sphingomonas (15, 19, 30*), *Massilia*, and *Arenimonas* (9, 62, 63), common inhabitants of ESSD and adapted to cold and harsh environmental conditions. In particular, *Polaromonas* species have been reported to be abundant in supraglacial debris in the Italian Alps (64) and among the weathering-associated bacteria from a Swiss glacier forefield (65). These observations highlight the versatility of bacteria members of these genera across different soil properties. Among the most abundant fungal core OTUs, we detected members of the *Ascomycota* and *Basidiomycota*, which can occur as hyphomycetes or as yeasts. These are globally widespread in the cryosphere, especially in barren soils near the glacier terminus (28, 30). Members of the genus *Tetracladium* are potential plant endophytes dispersing as aquatic hyphomycetes, a group of fungi often reported from glacial forefields worldwide (28, 66, 67). *Lycoperdon* spp., *Plenodomus biglobosus*, *Mycosphaerella (Davidiella) tassiana*, *Phenoliferia psychrophenolica*, and *Ganoderma* spp. have not been detected in this habitat before. Although members of the genera *Phenoliferia* (28) and *Ganoderma* (67) have been observed in glacial systems, the uniqueness of the species detected suggests a preferential habitat typical of our sites.

Taken together, the core and non-core OTUs followed distinct ecological patterns (Table S6). The strong A-O relation agrees with the neutral model predictions (NCM), which suggest that the majority of OTUs is as widespread as expected from their abundances (Fig. 5A; Table S6). The neutrally distributed OTUs are less likely to be specifically adapted to the habitat, although they may be just as functionally important as other OTUs, especially if abundant. Rather, the environment is not differentially selecting them, and thus, their distributions are the result of neutral dispersal and drift. Likewise, OTUs that are more widespread than expected (higher = selected for, e.g., many core OTUs) represent taxa that are specifically adapted to and selected by the soil habitat and are therefore likely to play important roles in the ecosystem. These results are consistent with the current theory of developmental processes (47), and they have also been observed in ESSD (e.g., 68). This confirms that the distinction between abundant and rare microbes and the study of individual populations, i.e., communities at the species level, can complement the overarching community framework and help to better understand the ecology and overall process of pedogenesis.

As a second aim, we asked if in calcareous glaciers there are bacterial-fungal associations that are selected for by the habitat. For this, we inferred association networks between bacterial and fungal OTUs (nodes) in each glacier forefield, and we searched for association pairs detected in all locations. This is one of the few studies to consider both microbial groups in a cohesive network, which allows us to capture higher-order interactions. At our sites, microbial association networks were very dense, thereby underlining the high frequency of associations among microbes in calcareous bedrock that might be of ecological relevance. Although the comparison of microbial networks derived from different analytical tools is not ideal (69), our results are consistent with the high network densities observed for ESSD in non-calcareous ecosystems during snow-free periods (16, 18). Importantly, we found that bacterial-fungal associations were very frequent (30%), and that fungal nodes had the highest degrees (Fig. S9), even though the networks contained much more bacterial than fungal nodes (Table 2). These results contrast with other reports that inter-group associations are less frequent than intra-group associations (15, 20), and that fungi were either isolated and ultra-peripheral nodes (19), or had the lowest degrees (18, 23). In any case, our results underline the high relevance of fungi for ESSD. While this may suggest differences in microbial interactions depending on the parent material, it also supports that these differences may be due to seasonality. Dresch et al. (28) reported that, on a similar glacier forefield site with carbonate minerals and high soil pH, seasonal shifts in microbial community from winter to summer started with a delay of at least 2 weeks after snowmelt. This delay is probably due to comparatively slow changes in soil properties (water content, temperature, and nutrient availability) after snowmelt. As we have comparatively excluded traces of microbes deriving from the snow (9, 44, 45), our sampling may be more representative of the microbial community present in snow-covered primary successional soil than in such soil under summer conditions. This is also supported by many of the abundant taxa detected (Fig. 3E and F), which are typical of winter soil fungal communities, but not of snow-associated microbes.

Sampling timing was often the most important factor explaining differences in microbial composition, even more important than vegetation type, habitat, and temperature (69). Fungi are usually better adapted to low-temperature conditions than bacteria (70, 71) and were reported to have increased abundance and biomass in Alpine habitats during winter (72, 73). Moreover, fungal growth was demonstrated in the frozen soil (74) and under the snow on a glacier forefield (28) in two Alpine sites with carbonate minerals and high soil pH. Altogether, we speculate that, under these conditions, fungi might fulfill key roles in the ecosystem, which might also explain their high connectivity in the inferred networks. Part of their ecological function may have a physical background: fungal hyphae elongate, branch, and move through the soil space. Their hyphal networks can provide a liquid layer of water (75, 76) necessary for bacterial mobility (i.e., bacterial highways [77]). The excretion of enzymes on the hyphal tips provides nutrients for associated microorganisms and may be connected to soil nutrient cycling. At later stages within the snow-free season, drought stress, high UV radiation, and large daily temperature fluctuations become limiting factors for fungal growth in these almost vegetation-free locations, thereby limiting fungal hyphal impact. However, this has not been experimentally tested in the field.

One could argue that the high density predicted by network inference does not accurately reflect microbial associations in ESSD of glacier forefields, and that a high number of false associations might have been predicted, or that (artificial) thresholds applied might have inflated the prediction. We agree that wet lab experiments testing the predicted associations, as well as robust and representative benchmarking studies for network inference, are desperately needed. However, we are convinced that network inference, which is widely applied, gives reasonable predictions of the microbial associations expected in the ecosystem modeled, especially given the relatively high number of biological replicates and samples used in this study.

Following the former observations, we searched for conserved pairwise associations across the four glacier forefields. Given the high microbial diversity found in these locations, no common associations could be expected (*P* = 0.469). The fact that we did not find common bacterial-fungal associations across the four networks implies that this habitat does not select for specific bacterial-fungal associations. Therefore, in these ecosystems, microbial associations are driven by neutral processes of dispersion and drift. Despite this randomness, many associations were estimated. This suggests an overall quite high degree of promiscuity and a relatively high level of opportunism among association partners.

It is important to note that this does not imply the irrelevance of bacterial-fungal associations. On the contrary, the dense networks and theoretical frameworks support their importance. Selected-for OTUs dominated the networks, were well integrated (Fig. S13), and equally paired with selected-against or neutral OTUs (Fig. 5B). This underlines that microbial associations might, to some degree, determine the community assembly. The ecological consequences of microbial associations generated by stochastic factors are many-fold. Many factors affect how microbial communities develop during the progress of pedogenesis. These include short-term disturbances (e.g., avalanches, landslides) and long-term climate change. All these factors can shift the community in unpredictable ways depending on the outcome of primary microbial assembly, drift, and selective pressures (abiotic and biotic).

Further, the NTI distribution indicates phylogenetic clustering of both bacterial and fungal communities, suggesting an environmental selection upon microbial taxa based on specific sets of phylogenetically conserved traits (homogeneous selection) (Fig. 4C and D). This selection is likely shaped by the very high local heterogeneity, providing a variety of different niches and microbial interactions or interaction opportunities. Under the current theoretical framework, newly available niches are randomly colonized by members that are functionally trait equivalent in their ability to disperse to and colonize a particular niche (9). Therefore, the community composition is determined by whichever suitable species happens to arrive on location first (lottery model) (9, 76).

However, dispersal processes are rarely purely stochastic. They are rather a combination of dispersal (i.e., movement through space) and establishment mechanisms, and highly dependent on the specific species traits, active status of microorganisms (deterministic factors), and interaction options. Many studies still treat dispersal as being neutral, because it is difficult for field studies to identify dispersal traits, link dispersal traits to community structure patterns, or assess dispersal processes and rates (78). Today, it is well recognized that microorganisms show strong biogeographical patterns, which is evidence for dispersal limitation (13). ESSD in glacier forefields are very heterogeneous, extremely selective environments: they are usually geographically isolated, nutrient-limited, and harsh (e.g., many freeze-thaw cycles). Thus, the microbial communities detected may have already been pre-selected, either during arrival or by their capability to establish, justifying the phylogenetic patterns observed in this study (Fig. 4C and D). Another hypothesis is that the phylogenetic clustering we observed is caused by the selective pressures that snow cover may impose on the microbial species in the soil during winter. Many physicochemical properties were measured here, including the ones which are commonly considered. Still, we only detected an insignificant influence of both spatial distance and environmental variables. Therefore, future research should focus more on the influence of seasonality shaping the microbial communities in these habitats.

Then, once within a specific niche, different organisms perform substrate degradation by “putting in common” (due to leakage, excretion, lysis, extracellular hydrolyzation) energy-rich metabolic intermediates (79–81). This sustains the growth of many other species (82) (79) who either have unique patterns of metabolite consumption, in order to minimize interspecific competition (83), or co-exist by exchanging mutually beneficial, essential metabolites (84). In this scenario, fungi can be expected to fulfill functional roles possibly regarding C-mobilization/acquisition and promiscuity toward bacterial associations (high connectivity), whereas bacteria fulfill specific sets of functions (e.g., rock weathering) that may be directed by abiotic soil characteristics or dense biotic interactions (26). Further experimental studies applying metagenomics, and especially metatranscriptomics, at the microscale level are needed to discriminate whether interactions *per se* increase the chances of interactive partners to survive (higher = selected for), or whether selected-for OTUs may be better located in the soil matrix, attached to substrates or other organisms (e.g., lichens), where interactions between fungi and bacteria are more likely to occur.

### Conclusions

Glacier forefields with calcareous bedrock harbor a diverse community of bacteria and fungi. They associate in dense microbial networks where fungi, although less in count, were more interconnected than bacteria. This suggests that the ecological role of fungi may be underestimated in this habitat.

In our comparative study across four calcareous glacier forefields in the Alps, we found that certain microbes (i.e., the core microbiome and selected-for OTUs) are more successful colonizers than others. Discriminating between abundant and rare microbes and studying individual populations, i.e., communities at species level, will help to better determine the dispersal and establishment success of microbial populations based on their specific traits and functional potential. This will complement the existing ecological framework and allow for a better understanding of the overall process of pedogenesis.

Our results emphasize the role of microbial associations in explaining community assembly. We show that in the early stages of soil development, neutral processes of dispersal and drift likely shaped the high diversity and number of microbial associations, thereby leading to opportunistic associations between microbes. These could further determine community assembly and promote the heterogeneity of the overall microbial composition. This important, potentially determining, stochastic component should be further explored under existing and new theoretical frameworks, because it may have profound implications for understanding the development of ecological systems, especially in future climatic scenarios.

## Data Availability

The datasets generated during the current study are available in the Sequence Read Archive (SRA) of the National Center for Biotechnology Information repository, under the accession number PRJNA1023863. R scripts are available under the Github repository: https://github.com/edoardo94psg/MICINSNOW.

## SUPPLEMENTAL MATERIAL

File S1a aem.00302-25-s0001.xlsx OTU tables with taxonomy and sequences for 16S across the four glaciers.

File S1b aem.00302-25-s0002.xlsx OTU tables with taxonomy and sequences for ITS across the four glaciers.

File S2 aem.00302-25-s0003.xlsx Bacterial and fungal OTUs of the core microbiome, with their relative abundance, taxonomic annotation, and sequence.

File S3 aem.00302-25-s0004.xlsx Pairwise associations between bacteria and fungi at the OTU level, with their taxonomic annotation, and relative abundance of the OTUs.

File S4 aem.00302-25-s0005.xlsx List of enzymes whose activity was determined, with buffers and substrates used for determination.

Supplementary Material and Methods aem.00302-25-s0006.pdf Figures S1 to S13; Tables S1 to S6.

ASM does not own the copyrights to Supplemental Material that may be linked to, or accessed through, an article. The authors have granted ASM a non-exclusive, world-wide license to publish the Supplemental Material files. Please contact the corresponding author directly for reuse.
