# Supplementary material for: Determinism and stochasticity drive microbial community assembly and microbial interactions in calcareous glacier forefields
Source: Appl Environ Microbiol. 2025 May 15;91(6):e00302-25. doi: 10.1128/aem.00302-25 (PMC12175511; doi:10.1128/aem.00302-25)
Supplement: Supplementary Material and Methods — Figures S1 to S13; Tables S1 to S6. [file aem.00302-25-s0006.pdf]

Determinism and stochasticity drive microbial dispersal and microbial interactions in calcareous glacier forefields

Index Supplementary Information

1) Supplementary Figures (pages 2 – 15)
2) Supplementary Tables (pages 16 – 21)
3) Supplementary excel file tables (page 22)
4) Supplementary Materials and Methods (pages 23 – 28)

Supplementary Figures

Figure S 1 Geographical locations of the four glaciers investigated in this study across the Alpine range. The sampling location is marked with a red star within each glacier picture to show the overview of the site in comparison with the ice.

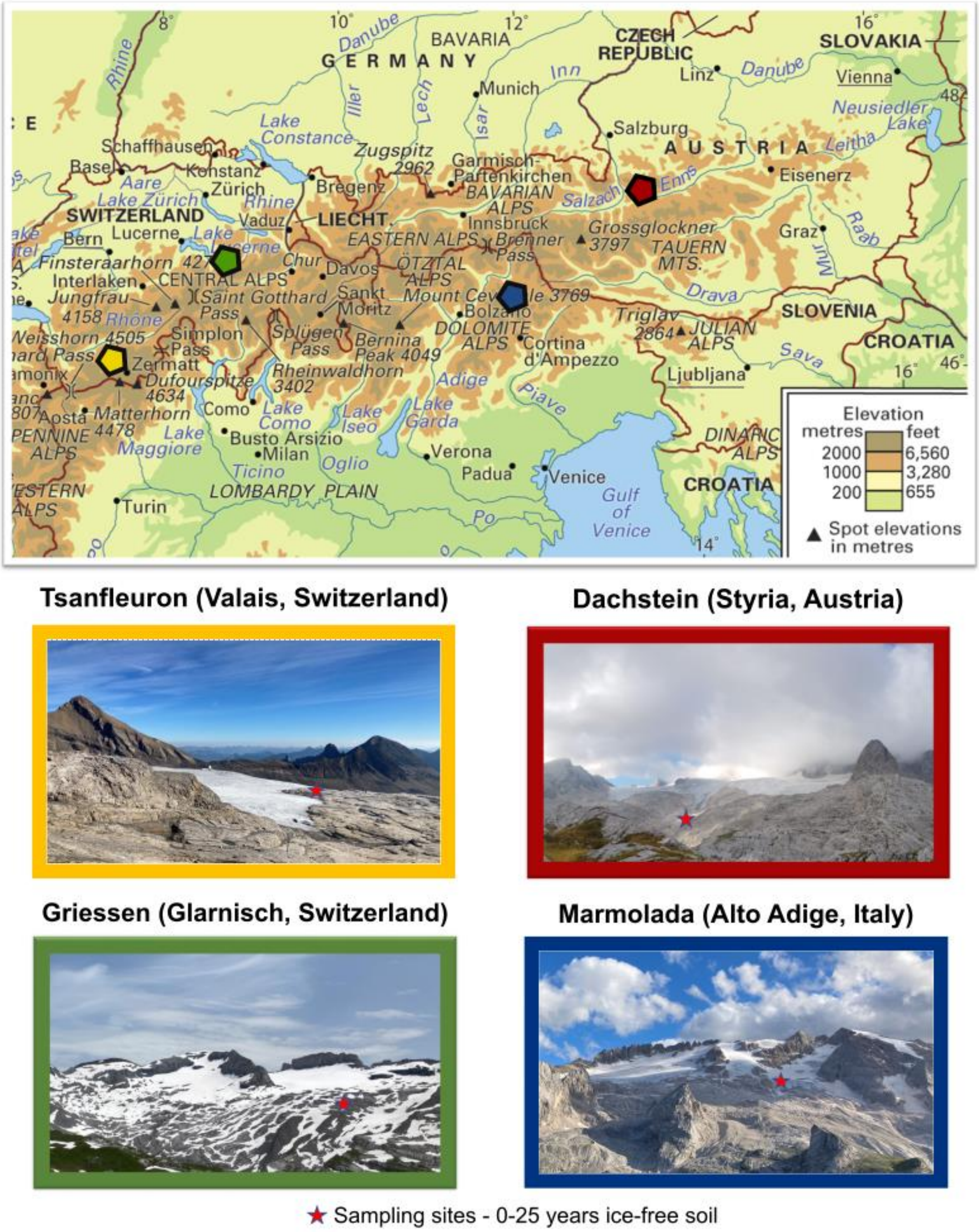

Figure S 2 Sampling site locations on glacier topographical maps. The glacier ice is shown in light blue. Scattered blue lines show approximate ice limit during sampling campaign in 2021.

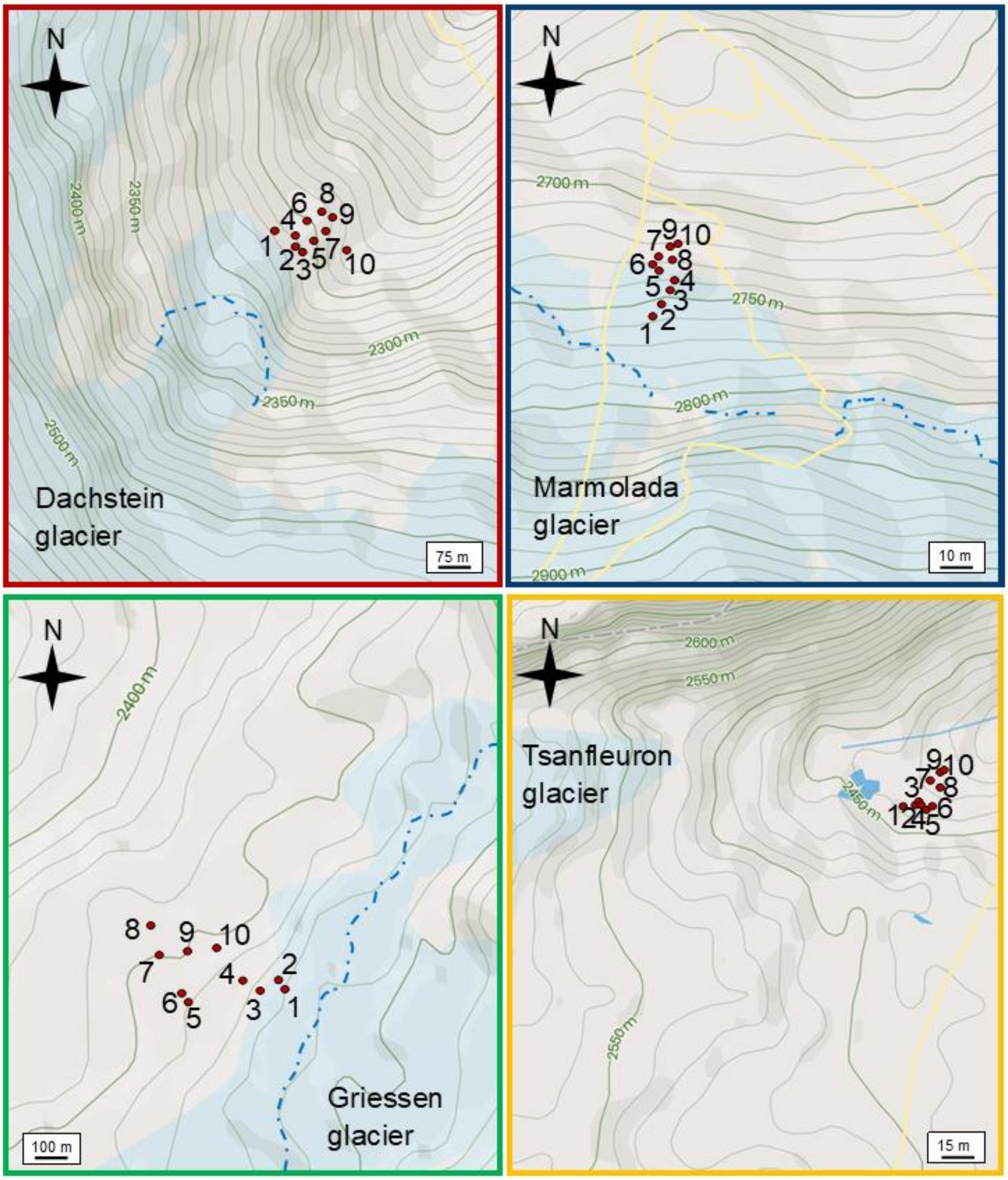

Figure S 3 Environmental factors characterizing the different glaciers (sample n = 10). Horizontal lines represent the median while the boxes represent the inter-quartile range of the first and third quartiles. The vertical lines (whiskers) represent the maximal and minimal values.

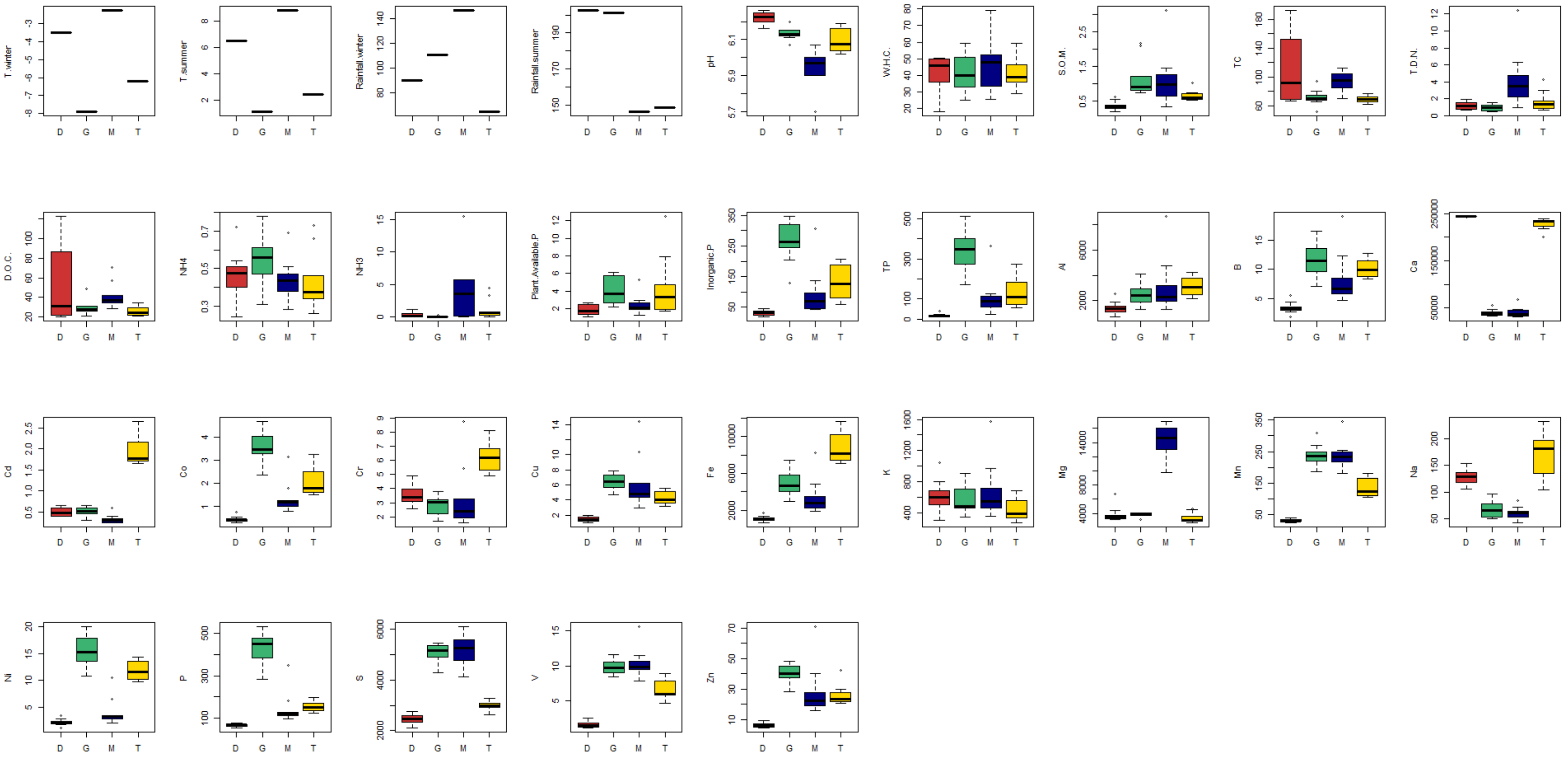

Figure S 4 Overview of sequencing depth for each glacier of bacterial and fungal datasets after quality filtering; Horizontal lines represent the median while the boxes represent the inter-quartile range of the first and third quartiles. The vertical lines (whiskers) represent the maximal and minimal values. Points within each boxplot represent the means (n=50, D; n=47, G; n=48, M; n=47, T). Letters indicate differences between individual means assessed with Kruskal-Wallis ($p_{bacteria}$ = 0.001; p_{fungi} = 0.004). Note that the y-axis scales are different for each plot.

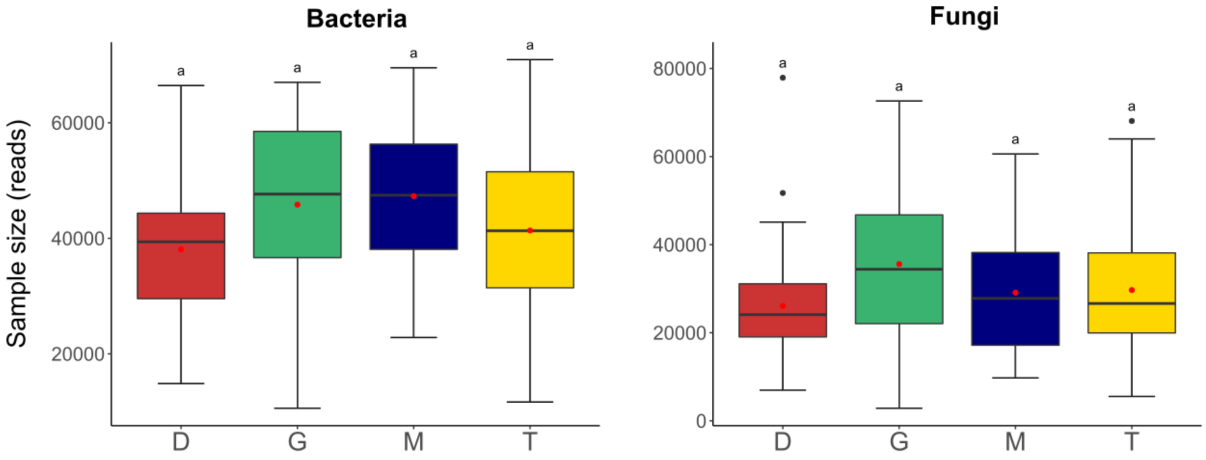

Figure S 5 Rarefaction curves for bacterial and fungal communities generated for each glacier. Each line is a subsample and the labelled is included. Note that the y-axis scales are different for each plot.

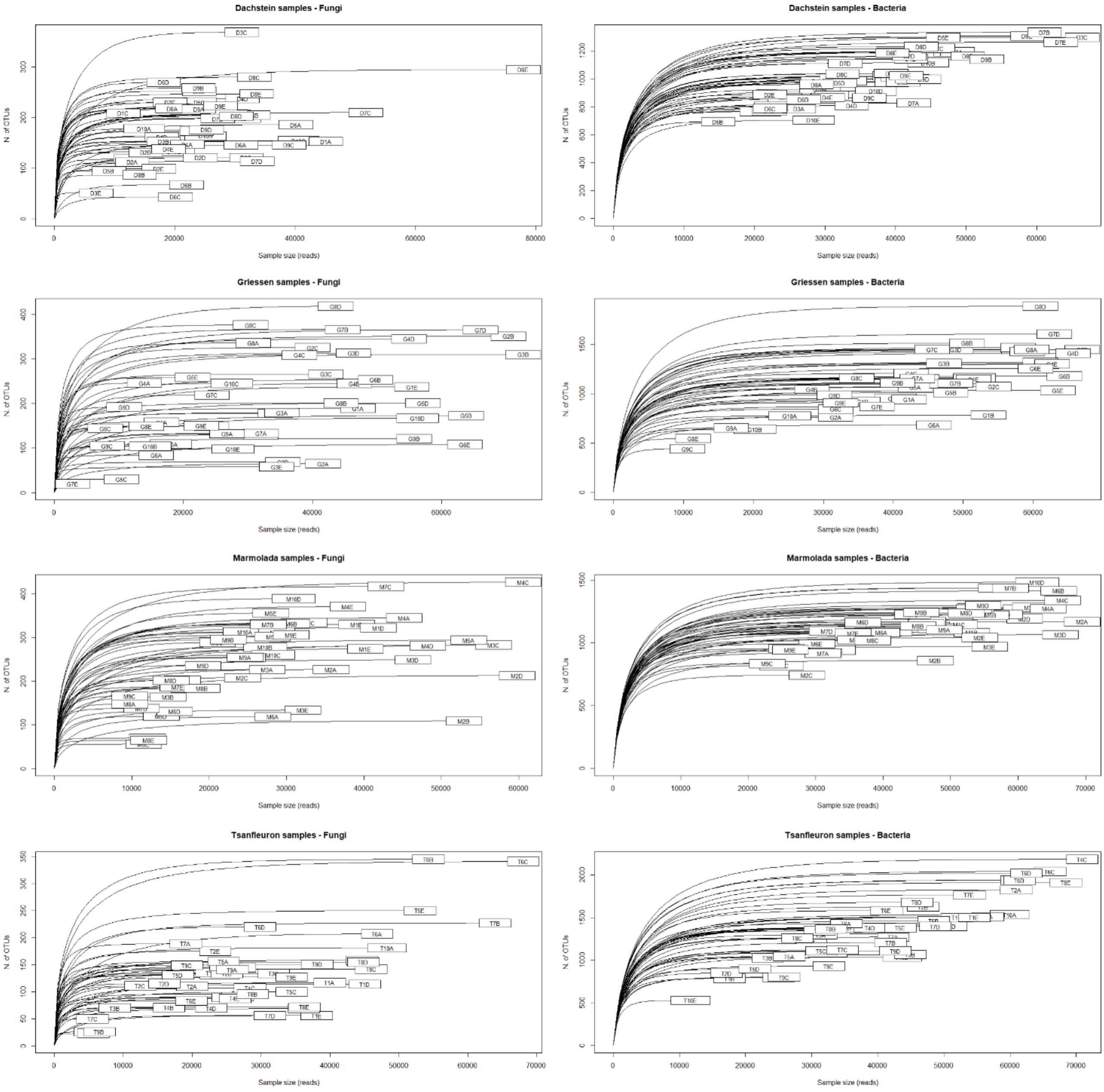

Figure S 6 The thresholds for the core community of bacteria and fungi present in a minimum of 10 and 5 samples within each glacier (dashed lines), respectively, were selected based on the distribution of sequencing reads (count) detected for each glacier. Consequently, the bacterial OTUs sets accounted for 27.7%, 24.3%, 29.9%, and 25.7% of the total reads for the D, G, M, and T forefields, respectively. The fungal OTU sets accounted for 20.3%, 19.0%, 21.5%, and 21.9% of the total reads for the D, G, M, and T forefields, respectively.

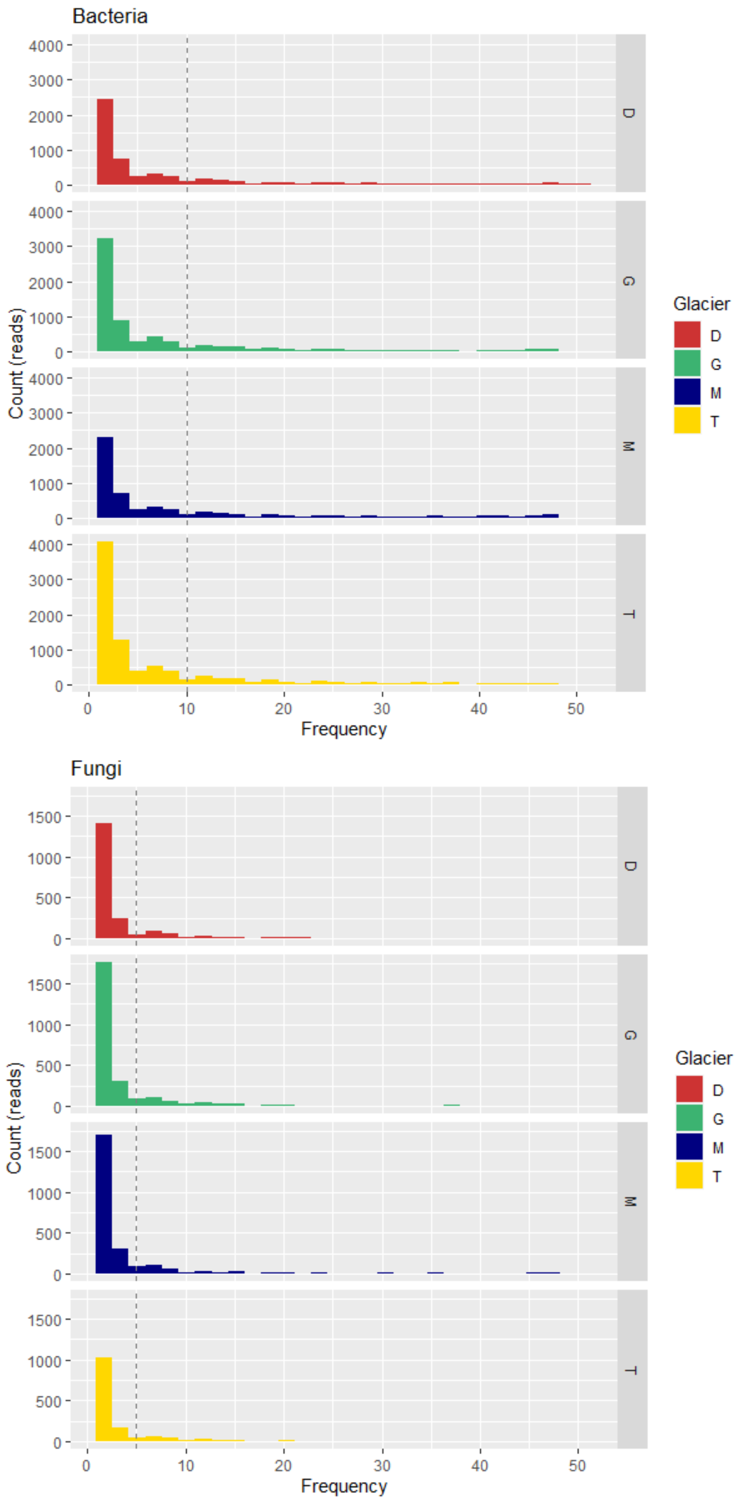

Figure S 7 Differences in α–diversity indices of the most abundant bacterial and fungal among glaciers. Horizontal lines represent the median while the boxes represent the inter-quartile range of the first and third quartiles. The vertical lines (whiskers) represent the maximal and minimal values. Points within each boxplot represent the means (n=50, D; n=47, G; n=48, M; n=47, T). Letters indicate differences between individual means assessed with Tukey HSD post-hoc tests. Note that the y-axis scales are different for each plot.

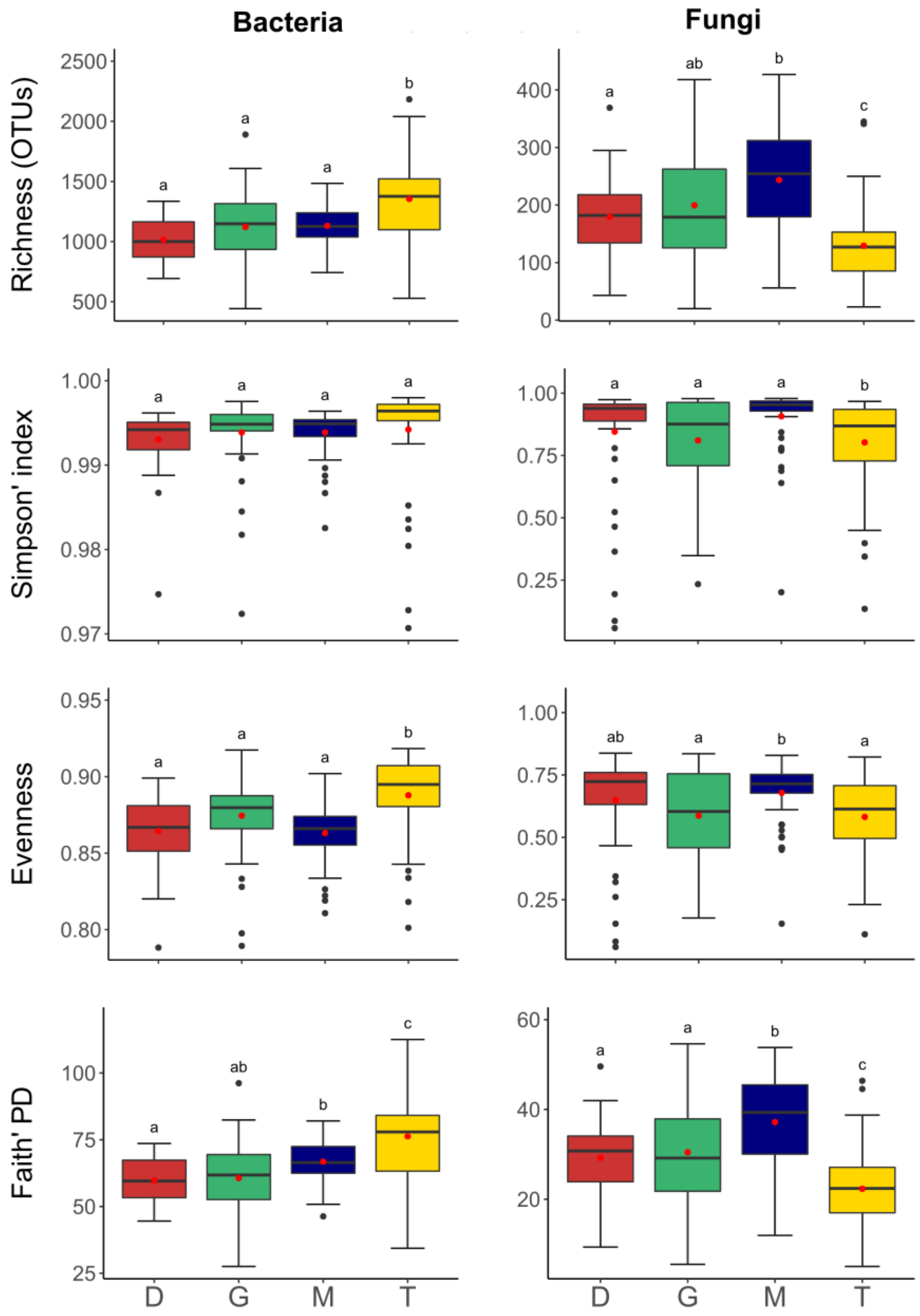

Figure S 8 Abundance-occurrence relationship for bacterial and fungal communities in each of the four glaciers. The correlation coefficients and p-values obtained in the Spearman correlation analysis are presented in each plot. OTUs belonging to the core microbiome are coloured in black in all plots.

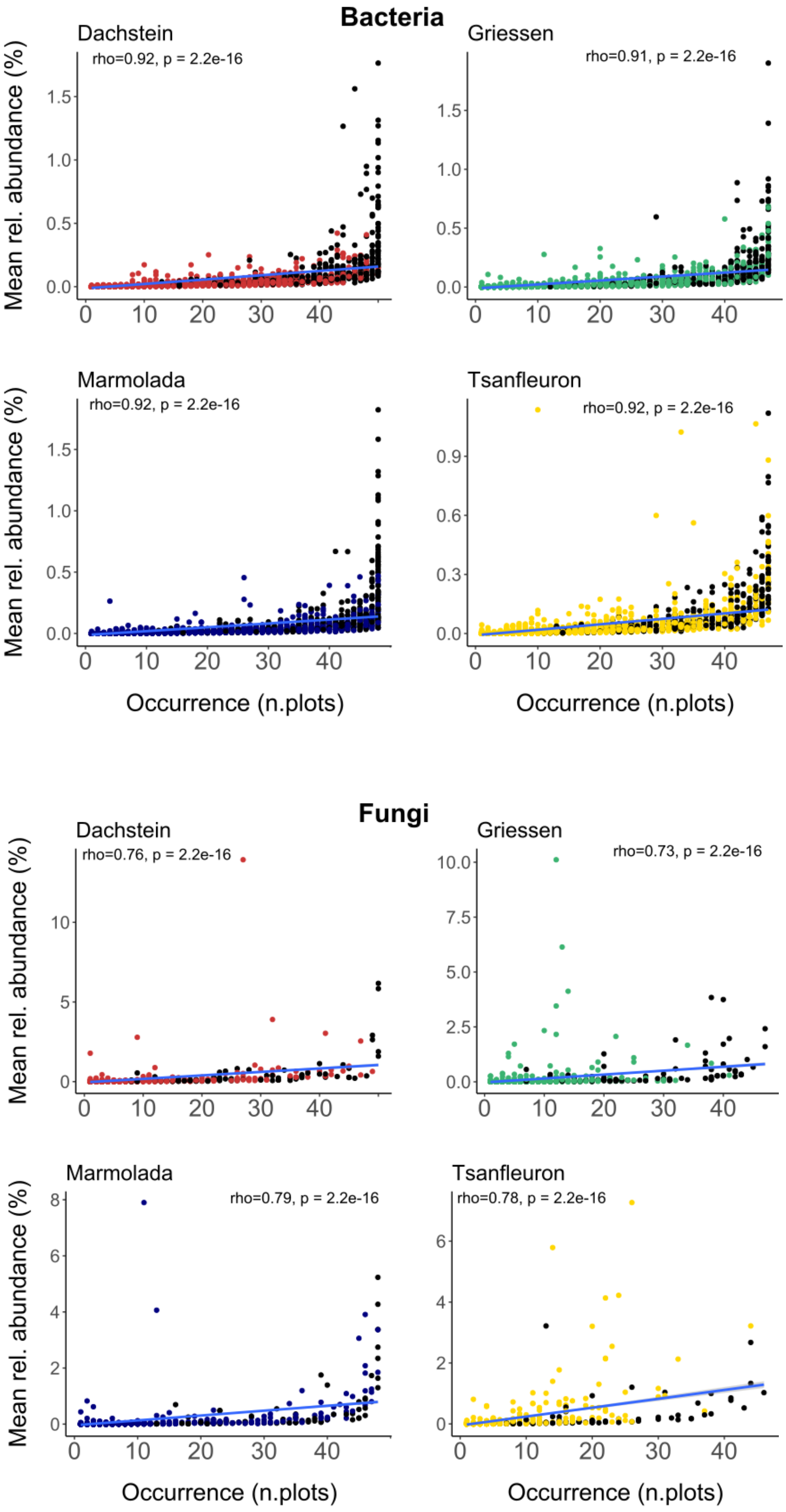

Figure S 9 Differences in edge numbers of the bacterial and fungal nodes among glacier networks. Horizontal lines represent the median while the boxes represent the inter-quartile range of the first and third quartiles. The vertical lines (whiskers) represent the maximal and minimal values. Points within each boxplot represent the means (n = n. of nodes). Letters indicate differences (ANOVA = bacterial $F_{3,6701} = 285.5$, $p<0.001$; fungal $F_{3,1656} = 28.85$, $p<0.001$) between individual means assessed with Tukey HSD post-hoc tests. Note that the y-axis scales are different for each plot.

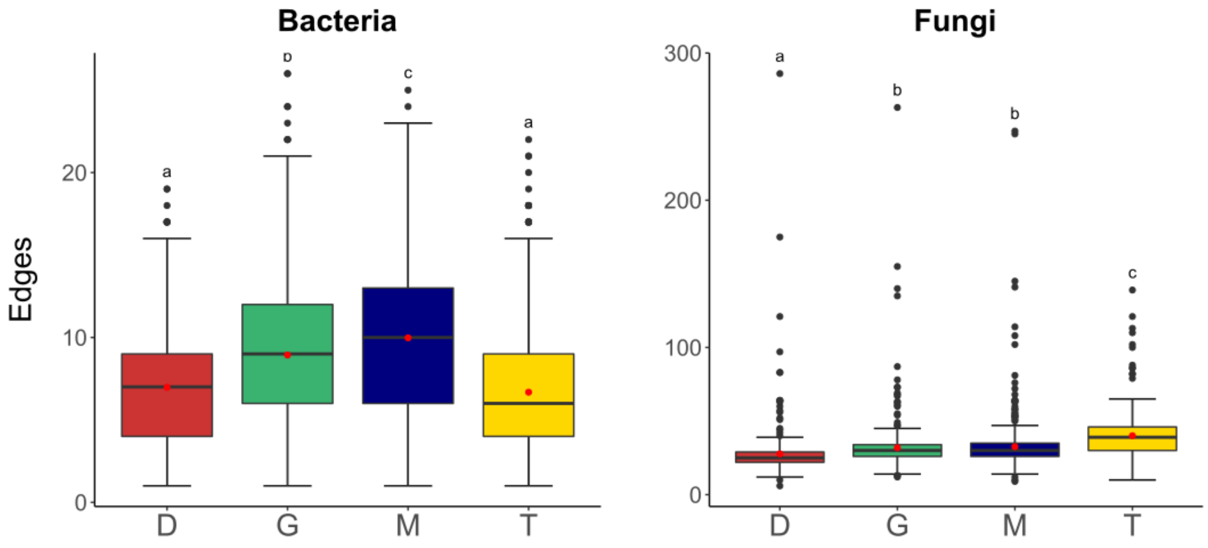

Figure S 10 Statistical differences between number of node' edges and bacterial and fungal nodes belonging to the core microbiome (core) or not (unique) in the glacier forefield networks. Horizontal lines represent the median while the boxes represent the inter-quartile range of the first and third quartiles. The vertical lines (whiskers) represent the maximal and minimal values. Letters indicate differences tested by Wilcoxon test between individual means. Note that the y-axis scales are different for each plot.

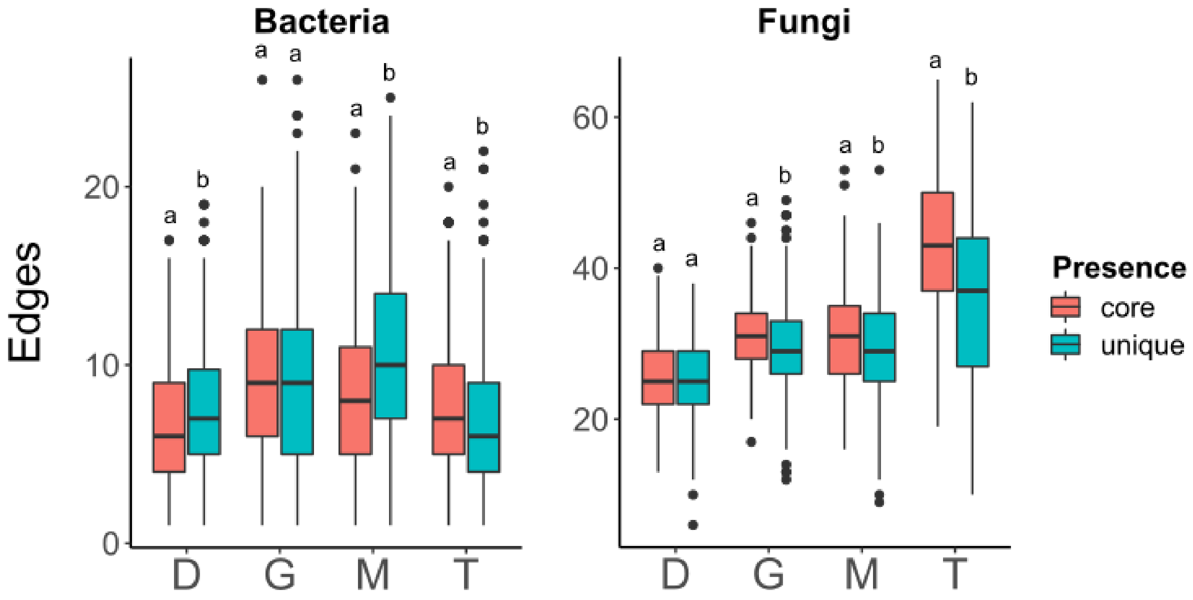

Figure S 11 Heatmap showing glaciers clusters based on shared pairwise bacterial-fungal associations at the genus level. Presence stands for how many times a pairwise association occurred in a glacier. Frequency stands for how many times a pairwise association occurred among the four glaciers, that is a pairwise association of frequency four, appeared in all four glaciers.

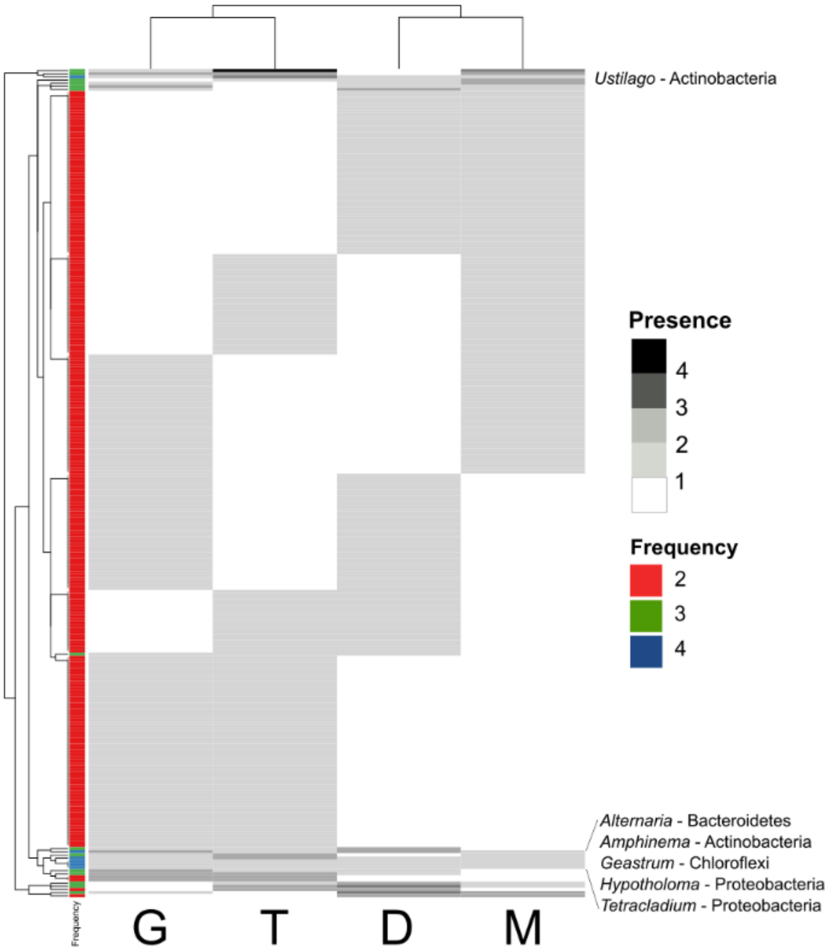

Figure S 12 Relationships among modules from the four bacterial-fungal association networks calculated for the glaciers under study. (a) Fluvial diagrams compare modules (numbered rectangles) between two glaciers by drawing a line between same OTUs in different glaciers. Colours are based on the clusters of the glacier on the left-hand side of the plots. (b) Dendrogram showing hierarchical cluster analysis (UPGMA) of modules among the four glacier networks. The analysis was based on the OTUs belonging to each module. Letters D, G, M, T refers to glaciers Dachstein, Griessen, Marmolada, Tsanfleuron, respectively. Clusters are coded by glacier name and a number.

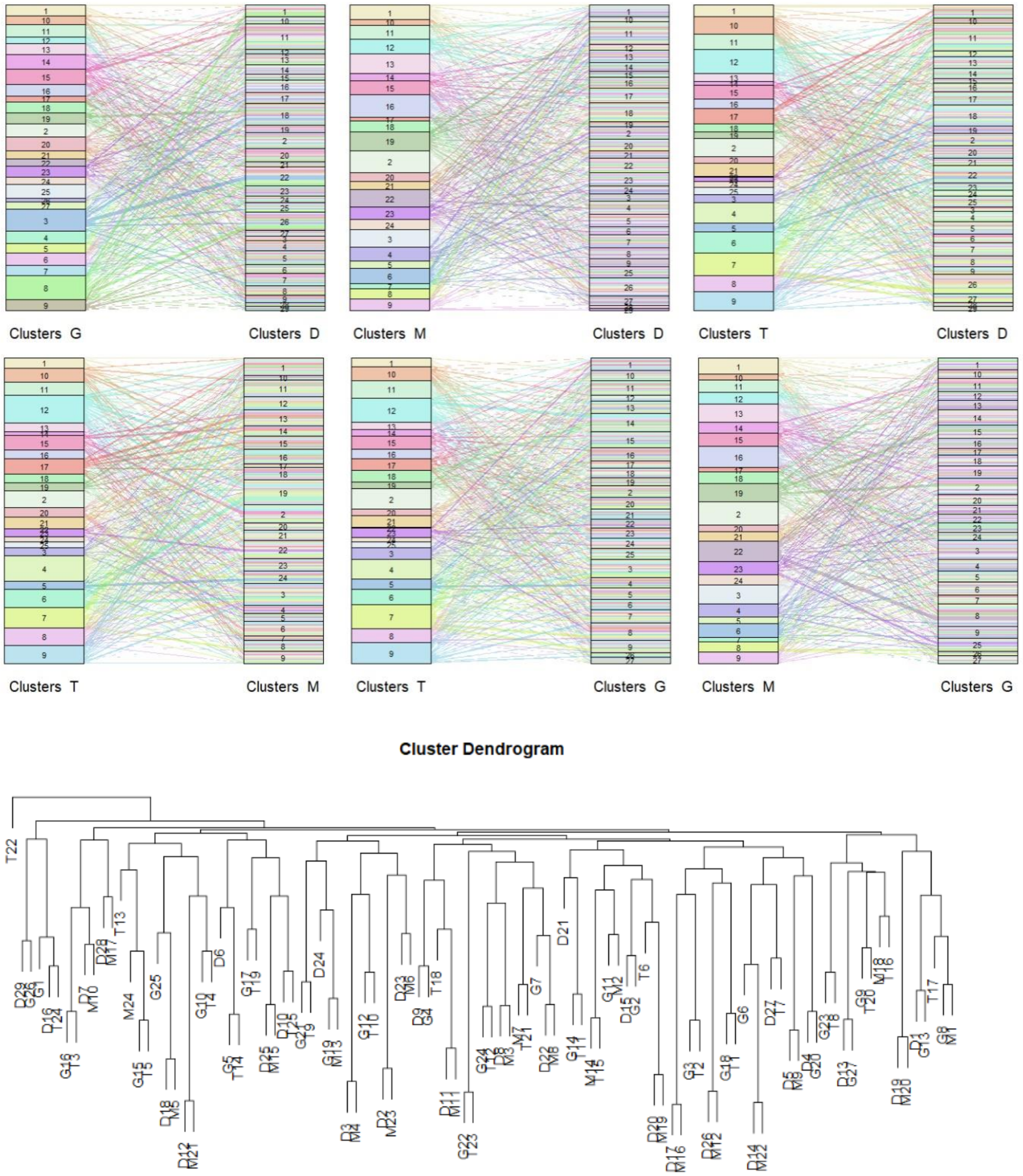

Figure S 13 Statistical differences between number of node' edges and Sloan Neutral Contribution Model (NCM) in the glacier forefield networks. Horizontal lines represent the median while the boxes represent the inter-quartile range of the first and third quartiles. The vertical lines (whiskers) represent the maximal and minimal values. Letters indicate differences between contributions within each glacier assessed with Dunn tests after Kruskal-Wallis tests. Note that the y-axis scales are different for each plot.

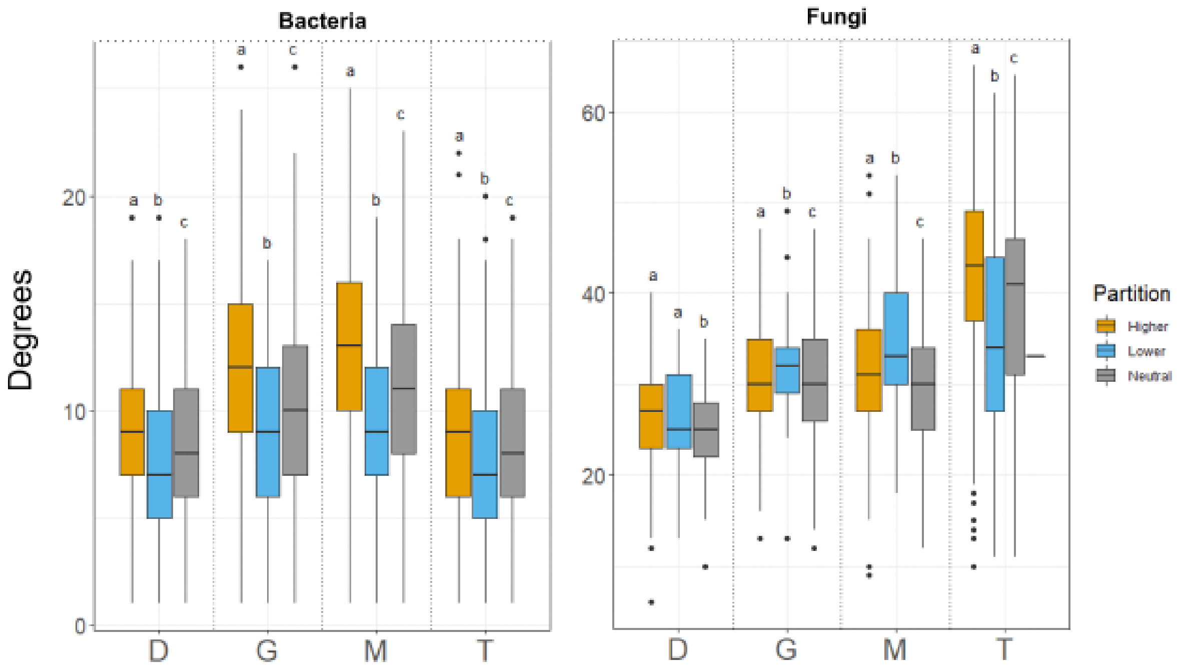

Supplementary Tables

Table S 1 Coordinates for each sampling site within the four glaciers.

Sample Site	Dachstein	Griessen	Marmolada	Tsanfleuron
**1**	47.4875639, 13.6184222	46.8422, 8.490461	46.441896, 11.859656	46.324972222, 7.250638889
**2**	47.4875333, 13.6192611	46.84227, 8.490443	46.44198, 11.85965	46.324972222, 7.250666667
**3**	47.4876222, 13.6194083	46.84198, 8.489745	46.442128, 11.859677	46.324972222, 7.250666667
**4**	47.4876778, 13.6190694	46.842167, 8.489517	46.442224, 11.859714	46.324972222, 7.250694444
**5**	47.4877639, 13.619525	46.842149, 8.48867	46.44237, 11.859503	46.324916667, 7.250777778
**6**	47.4879694, 13.6193083	46.842061, 8.488705	46.442455, 11.859529	46.324888889, 7.250805556
**7**	47.487722, 13.61975	46.842709, 8.488714	46.442532, 11.859596	46.32525, 7.251388889
**8**	47.4879194, 13.6197167	46.843005, 8.488532	46.442445, 11.859812	46.325361111, 7.251333333
**9**	47.488, 13.619889	46.842738, 8.489132	46.442549, 11.859841	46.325472222, 7.251416667
**10**	47.4881833, 13.6197278	46.842835, 8.489626	46.442569, 11.859925	46.3255, 7.251444444

Table S 2 Statistical differences (F-values) and means (± s.d., n = 10) of environmental factors characterizing the different glaciers investigated. Letters indicate differences between individual means assessed with Tukey HSD post-hoc tests.

		Dachstein	Griessen	Marmolada	Tsanfleuron	$F_{3,36}$§
**pH**		6.2 ± 0^{a***}	6.1 ± 0^{ac***}	6 ± 0.1^{b***}	6.1 ± 0.1^{c***}	29.6
**WHC**		42.1 ± 10^{NS}	41 ± 11.3^{NS}	43.4 ± 11.5^{NS}	41.2 ± 8.7^{NS}	0.6
**SOM**		0.4 ± 0.1^{a***}	1.1 ± 0.5^{bc***}	0.9 ± 0.3^{c***}	0.6 ± 0.2^{abc***}	6.6
**TC**		107.7 ± 44.6^{a**}	71.4 ± 10.8^{ab**}	93.6 ± 14.6^{ab**}	69.1 ± 5.1^{b**}	5.9
**DOC**		51.6 ± 42^{NS}	29.4 ± 7.4^{NS}	41.7 ± 13.7^{NS}	26 ± 4.6^{NS}	7.4
**TDN**		1.2 ± 0.5^{a***}	1 ± 0.4^{a***}	3.3 ± 1.7^{b***}	1.6 ± 1.2^{a***}	2.7
**NO_3^-**		0.3 ± 0.4^{ab**}	0.1 ± 0.1^{b**}	2.7 ± 2.5^{a**}	1.2 ± 1.5^{ab**}	1.2
**NH_4^+**		0.5 ± 0.1^{NS}	0.5 ± 0.1^{NS}	0.4 ± 0.1^{NS}	0.4 ± 0.2^{NS}	5.3
**Plant Available P**		1.9 ± 0.6^{a**}	4.1 ± 1.5^{b**}	2.1 ± 0.6^{a**}	4.4 ± 3.4^{b**}	4
**PO_4^{3-}**		31 ± 8.5^{a***}	265.8 ± 65.1^{b***}	70.2 ± 31^{ac***}	134.2 ± 55.4^{c***}	28.6
**TP**		17.1 ± 9^{a***}	345.9 ± 105.6^{b***}	81.1 ± 33^{ad***}	131 ± 69.8^{cd***}	30.9
**Major**	**Ca**	243960 ± 767.7^{a***}	39575 ± 6771.2^{b***}	39722.2 ± 11357.2^{b***}	228190 ± 12016.8^{c***}	1656.6
	**Mg**	3827.5 ± 1071.4^{a***}	3835.5 ± 275.9^{a***}	14038.9 ± 2140.9^{b***}	3366 ± 733.9^{a***}	177.4
	**Fe**	1068 ± 288.3^{a***}	4853 ± 1334.9^{b***}	2836.7 ± 911.5^{b***}	8760 ± 1608.6^{c***}	51.4
	**S**	2457 ± 197.4^{a***}	5066 ± 423.2^{b***}	5255 ± 582.7^{b***}	2989 ± 181.5^{a***}	138.2
	**Al**	1432 ± 513.6^{a***}	2487.5 ± 828.1^{ab***}	2457.8 ± 1045.4^{b***}	3150 ± 724^{b***}	4
**Minor**	**K**	610.8 ± 206.5^{NS}	573.5 ± 189.9^{NS}	574.3 ± 192.7^{NS}	443.2 ± 137.1^{NS}	1.7
	**Na**	129.2 ± 14.3^{a***}	67.9 ± 15.9^{b***}	57.8 ± 8.7^{b***}	167.3 ± 41.5^{a***}	45
	**Mn**	31 ± 4.5^{a***}	239.4 ± 33.4^{b***}	224.4 ± 25.7^{b***}	131.9 ± 28.8^{a***}	97.9
	**P**	67.8 ± 6.8^{a***}	422.4 ± 82.5^{b***}	123.1 ± 25.1^{a***}	155.2 ± 24.5^{a***}	72.9
**Trace**	**Zn**	6.3 ± 1.4^{a***}	40 ± 6.2^{b***}	23.2 ± 7.3^{bc***}	25.8 ± 6.6^{c***}	21.6
	**B**	3.4 ± 1^{a***}	11.6 ± 2.7^{b***}	7 ± 2.3^{c***}	10.2 ± 1.5^{bc***}	16.9
	**Ni**	2.2 ± 0.6^{a***}	15.4 ± 3^{b***}	3.4 ± 1.3^{a***}	11.8 ± 1.7^{c***}	81.6
	**V**	1.6 ± 0.4^{a***}	9.9 ± 1^{b***}	9.8 ± 1^{b***}	6.4 ± 1.4^{c***}	87.7
	**Cu**	1.5 ± 0.3^{a***}	6.4 ± 1.1^{b***}	5.2 ± 2.1^{b***}	4.2 ± 0.8^{ab***}	14.3
**Other**	**Cd**	0.5 ± 0.1^{a***}	0.5 ± 0.1^{a***}	0.3 ± 0.1^{a***}	1.9 ± 0.3^{b***}	159.6
	**Co**	0.5 ± 0.1^{a***}	3.6 ± 0.7^{b***}	1.2 ± 0.3^{c***}	2.1 ± 0.6^{c***}	49.4
	**Cr**	3.5 ± 0.7^{a***}	2.8 ± 0.7^{a***}	2.6 ± 1.2^{a***}	6.2 ± 1^{b***}	13.5

Abbreviations: WHC = water holding capacity; DOC = dissolved organic carbon; TDN = total dissolved nitrogen; NO_3^- = nitrate; NH_4^+ = ammonium; P = phosphorous; PO_4^{3-} = phosphate; TP = total phosphorous.
All units are in µg g^{-1} dry weight of soil; except pH, WHC (%), and SOM (%).
§Significance of tests were based on F-ratios where the indices are the degrees of freedom and error terms.
Significance levels: ***p<0.001; **p<0.01; *p<0.05. NS, nonsignificant.

Table S 3 Statistical differences (F-values) and means (± s.d., n=50, D; n=47, G; n=48, M; n=47, T) of α-diversity parameters of bacterial and fungal communities for the four different glaciers.

	**α-diversity**	**Dachstein**	**Griessen**	**Marmolada**	**Tsanfleuron**	$F_{3,188}$ §
**Bacteria**	*Richness*	1015.1 ± 172.6***	1121.2 ± 299.6***	1131.1 ± 165.2***	1354.7 ± 369.7***	18.23
	Shannon	6 ± 0.2***	6.1 ± 0.3***	6.1 ± 0.2***	6.4 ± 0.3***	27.03
	Simpson	1 ± 0 NS	1 ± 0 NS	1 ± 0 NS	1 ± 0 NS	0.76
	Evenness	0.9 ± 0***	0.9 ± 0***	0.9 ± 0***	0.9 ± 0***	14.62
	Faith' PD	59.8 ± 8.1***	60.6 ± 14.1***	66.8 ± 7.9***	76.3 ± 18.4***	24.3
**Fungi**	*Richness*	179.5 ± 65.1***	199.6 ± 101.9***	243.5 ± 93.5***	129.4 ± 69.1***	18.49
	Shannon	3.3 ± 1***	3 ± 1.1***	3.7 ± 0.7***	2.8 ± 0.9***	9.77
	Simpson	0.8 ± 0.2*	0.8 ± 0.2*	0.9 ± 0.1*	0.8 ± 0.2*	3.7
	Evenness	0.6 ± 0.2*	0.6 ± 0.2*	0.7 ± 0.1*	0.6 ± 0.2*	4.4
	Faith' PD	29.2 ± 8***	30.5 ± 12.1***	37.2 ± 10.9***	22.3 ± 9.1***	20.68

§Significance of tests were based on F-ratios where the indices are the degrees of freedom and error terms.
Significance levels: ***p<0.001; **p<0.01; *p<0.05. NS, nonsignificant

Table S 4 Statistical differences (F-values) and means (± s.d.; n=50, D; n=47, G; n=48, M; n=47, T) of enzymatic activities for the four different glaciers. The nutrient cycle in which the enzymes belong is also added. Letters indicate differences between individual means assessed with Tukey HSD post-hoc tests.

Nutrient cycle	Enzyme	Dachstein	Griessen	Marmolada	Tsanfleuron	$F_{3,175}$§
S	**aryS**	0.8 ± 0.9 ^{a***}	0.7 ± 1.2 ^{a***}	5.2 ± 5.3^{b***}	1 ± 1.3 ^{a***}	12.74
C	**betaG**	0.9 ± 0.7 ^{a***}	0.8 ± 0.5 ^{a***}	2.3 ± 2 ^{b***}	1 ± 1 ^{a***}	45.45
N	**chit**	0.9 ± 0.8 ^{a***}	0.7 ± 0.4 ^{a}	2.5 ± 3.1 ^{b}	0.9 ± 0.9 ^{a***}	19.69
	**Protease**	14.7 ± 10.4 ^{ab***}	10.3 ± 5 ^{a***}	18.9 ± 11.3 ^{b***}	11.1 ± 4.4 ^{a***}	13.8
	**leu**	6.4 ± 6.6 ^{a***}	5.1 ± 3.3 ^{a***}	22.8 ± 26 ^{b***}	3.9 ± 3.7 ^{a***}	16.37
P	**acP**	0.4 ± 1.4 ^{a***}	2.2 ± 2.2 ^{a***}	6.1 ± 6.3 ^{b***}	1.9 ± 2.2 ^{a***}	24.5
	**bisP**	3.8 ± 2.4 ^{a***}	3.9 ± 2.2 ^{a***}	6.9 ± 5.6 ^{b***}	3.8 ± 2.7 ^{a***}	34.9
	**alkP**	23.5 ± 14.6 ^{a***}	23.8 ± 13.8 ^{a***}	47.8 ± 45.8 ^{b***}	23 ± 15.6 ^{a***}	13.76
	**FDA**	1.2 ± 0.7 ^{a***}	1 ± 0.3 ^{a***}	1.7 ± 1.2 ^{b***}	1 ± 0.3 ^{a***}	15.43
	**Oxidase**	1.1 ± 1.7 ^{a***}	1.6 ± 1.7 ^{a***}	3.2 ± 7.2 ^{b***}	1.3 ± 2.2 ^{a***}	11.39

Enzyme abbreviations: aryS = arylsulfatase; betaG = beta-glucosidase; chit = chitinase; leu = leucine-aminopeptidase; acP = acid phosphomonoesterase; bisP = phosphodiesterase; alkP = alkaline phosphomonoesterase; FDA = fluorescein diacetate hydrolysis.

§Significance of tests were based on F-ratios where the indices are the degrees of freedom and error terms.

Significance levels: ***p<0.001; **p<0.01; *p<0.05.

Table S 5 Statistical differences (PERMANOVA) in β-diversity of bacterial and fungal communities between the glaciers considering the environmental factors.

	**Variance**	**$F_{7,30}$§**	**P**
**Bacterial OTUs**			
Plant.Available.P	0.035849	1.996705	0.025
pH	0.043239	2.408318	0.013
Ca	0.059212	3.297978	0.003
Cd	0.053783	2.995602	0.002
TC	0.086215	4.80203	0.001
T.D.N.	0.063475	3.535429	0.001
Inorganic.P	0.119611	6.662091	0.001
**Fungal OTUs**			
Cd	0.038758	1.667989	0.011
T.D.N.	0.039835	1.714311	0.006
Ca	0.045814	1.971637	0.002
pH	0.068512	2.948451	0.001
B	0.063511	2.733238	0.001

§Significance of tests were based on F-ratios where the indices are the degrees of freedom and error terms.
Abbreviations: TDN = total dissolved nitrogen; P = phosphorous; TC = total carbon; Ca = calcium; Cd = cadmium; B = boron.

Table S 6 Percentage of the OTU portion for bacterial and fungal communities estimated by the neutral processes based on the Sloan Neutral Contribution Model (NCM). Percentages are reported for each glacier forefield.

		Partition	Dachstein	Griessen	Marmolada	Tsanfleuron
**Overall**	**Bacteria**					
		**Higher**	37%	35%	37%	28%
		**Lower**	7%	7%	7%	6%
		**Neutral**	56%	58%	56%	66%
	**Fungi**					
		**Higher**	35%	29%	31%	37%
		**Lower**	2%	3%	2%	5%
		**Neutral**	63%	69%	67%	58%
**Unique**	**Bacteria**					
		**Higher**	36%	39%	37%	27%
		**Lower**	5%	6%	5%	5%
		**Neutral**	59%	71%	59%	69%
	**Fungi**					
		**Higher**	32%	26%	29%	33%
		**Lower**	2%	3%	2%	4%
		**Neutral**	66%	72%	69%	62%
**Core**	**Bacteria**					
		**Higher**	49%			
		**Lower**	29%			
		**Neutral**	22%			
	**Fungi**					
		**Higher**	81%			
		**Lower**	9%			
		**Neutral**	10%			

Supplementary file tables:

Excel file 1a_16S.otu_glaciers_taxonomy: OTU tables with taxonomy and sequences for 16S across the four glaciers.

Excel file 1b_ITS.otu_glaciers_taxonomy: OTU tables with taxonomy and sequences for ITS across the four glaciers.

Excel files 2_Core_microbiome: Bacterial and fungal OTUs of the core microbiome, with their relative abundance, taxonomic annotation and sequence.

Excel file 3_Pairwise-association-network-bf: Pair-wise associations between bacteria and fungi at the OTU level with their taxonomic annotation, and relative abundance of the OTUs.

Excel file 4_Enzymes: List of enzyme whose activity was determined, with buffers and substrates used for determination.

Supplementary Material and Methods

Sampling procedure

For each plot, we selected safe-sites, that is small areas, concave surfaces, or little depressions on the terrain, often surrounded by big stones, that could ensure protection and stable mechanical conditions to the terrain from wind, stream of melting waters, snow dispatch, or sudden mudslides. Consequently, safe-sites are filled up of stone debris or mineral mud that become colonized by opportunistic pioneer plants (Table 1) and undergo early stages of pedogenesis.

Soil geochemistry

Soil pH was determined by equilibrating 5 g (fresh weight) of soil in 25 ml 0.01 M $CaCl_2$ for 1 h by shaking at room temperature. 2 h post-settling, the supernatants were analyzed using a pH meter (1). Plant-available phosphorous (plant available P; µg g-1 dry weight (dw)), total phosphate (TP; µg g-1 dw), and inorganic phosphorous (inorganic P; µg g-1 dw) were determined using the colorimetric molybdenum blue method as previously described (2).

Prior to the following analyses, soils samples were dried at 105 °C overnight. Dry weight was determined by loss of weight. Soil organic matter (SOM; %) was estimated by the loss of ignition method at 450 °C for 5 h (3) using 4 g soil dw. Water holding capacity (WHC; %) of soils was measured by pouring 50 ml of water into 25 g dw of soil contained in a paper filter funnel on top of a graduated cylinder. Water was left to percolate by gravity for 24 h and the WHC was calculated by dividing the volume of water retained by soil by its original dw.

Total carbon (TC; µg g-1 dw), total dissolved nitrogen (TDN, µg g-1 dw) and dissolved organic carbon (non-purgeable organic carbon, DOC; µg g-1 dw) were quantified on a TOC-L/TNM-L analyser (Shimadzu, Japan) after extracting 10 g fresh soil in 35 mL ultra-pure water by shaking for 1h at 150 rpm and filtering (Macherey & Nagel 615¼, 150 mm filter paper). Ammonium (NH_4^+-N; µg g-1 dw) and nitrate (NO_3^--N; µg g-1 dw) were determined with a Continuous Flow Analyzer (CFA, Skalar, Netherlands) based on a modified Berthelot and cadmium reduction method, respectively, after shaking 7.5 g fresh soil in 20 mL KCl [1 M] for 1h at 120 rpm followed by filtration (Macherey & Nagel 615¼, 150 mm filter paper).

Elemental concentrations (µg g-1) were determined by Inductively Coupled Plasma Optical Emission Spectroscopy (ICP-OES; Spectro Genesis FES, AMATEK Inc, Germany). Prior to measurements, samples were prepared by adding 1 g dw of ball-milled (Retsch S1 planetary) soil sieved with a 0.125 mm sieve into a Teflon vessel (Berghof Fluoroplastics, Germany). Subsequently 8 ml of HNO_3 (65%), was added to the vessels which were then shaken, hermetically sealed, and subjected to microwave assisted heating (SpeedWave, Berghof, Germany) at 180 °C for 40 min. Solutions were then filtered through a paper filter (Macherey-Nagel, Germany) and topped to 50 ml with ddH_2O. The detection limit reached was in the order of 1 µg ml-1.

Enzymatic activities

Enzyme activities were quantified from every subsamples in soil extracts following the procedure as previously described (4). Briefly, enzymes were extracted by heteromolecular

exchange using 2-mL Eppendorf tubes containing soil and a 3% lysozyme solution using a beating mill at 30 strokes s-1 for 3 min. The supernatant containing the desorbed enzymes was then centrifuged at 20,000 g for 3 min and 20 µL of extracts were pipetted in duplicate on 384-well microplates with 50 µL of appropriate buffer. After addition of specific substrate, enzyme activity was performed using a Synergy HT microplate reader (BIO- TEK). The list of enzyme activities determined, buffers and substrates used for determination is added in the Supplementary File - Enzymes.

Quantitation of microbial biomass was carried out as previously described (5). The procedure for double-strand DNA (dsDNA) extraction from soil is similar to that described above for enzymes, except for the buffer, being 0.12M, pH 7.8 sodium phosphate. Pico-green reagent (Thermo Fisher) was used to selectively quantify dsDNA in 80-fold diluted extracts. Microbial biomass was expressed as microgram of (dsDNA) .

Community profiling of bacteria and fungi

The primers used for sequencing were primer pair 515F (5′-GTGCCAGCMGCCGCGGTAA-3′) and 806R (5’- GGACTACHVGGGTWTCTAAT-3’) for amplification of the V4-16S rRNA region of bacteria (6) and the primer pair gITS7 (5’- GTGANTCATCGANTCTTTG3’) and ITS4 (5’- TCCTCCGCTTATTGATATGC-3’) targeting the ITS2 region of fungi (7).

Raw, demultiplexed 16S and ITS amplicon sequences were trimmed off primers, adapters and barcodes and quality filtered using cutadapt (cutadapt v1.18 (8)) prior to further processing. Low-quality tails of bacterial forward and reverse amplicons were trimmed to 220 and 200 bases, respectively. Then, amplicon sequence variants (ASVs) were inferred using DADA2 (9). First, sequences were filtered, settings were maximum number of Ns (maxN) = 0, maximum number of expected errors (maxEE) = (2, 2). After merging paired reads (mergePairs), chimeric sequences were removed (removeBimeraDenovo; default settings). Then, bacterial reads shorter than 240 bp or longer than 260 bp were removed. Finally, relative abundance tables were constructed. To this end, ASVs were clustered into operational taxonomic units (OTUs) with 97% identity (AlignSeqs, to align the sequences; IdClusters, to complete the clustering; DECIPHER package v2.26.0 (10)). Because OTU clusters may contain ASVs of different length, the longest ASV within each OTU cluster was selected as representative of the cluster. This allowed for retaining as much molecular information as possible for the taxonomical assignment. Thus, bacterial OTUs were taxonomically assigned up to genus level (assignTaxonomy; default settings). The Silva Project’s v132 preformatted release (11) was used for 16S rRNA annotation.

For fungal sequences, the same pipeline described above was used, with few differences. Namely, forward and reverse amplicons shorter than 50 bp were removed using (minLen) = 50. Filtering settings were maximum number of Ns (maxN) = 0, maximum number of expected errors (maxEE) = (3, 5). Fungal reads of all lengths were maintained for ASVs construction. As for the bacterial ASVs, fungal ASVs were clustered into OTUs with 97% identity. The taxonomic affiliation of each unique fungal OTU was obtained using UNITE Fungal ITS training set 10-05-2021 (12) with a confidence threshold of 80%.

Fungal taxa regularly exhibit exceptionally high variability in the ITS region, potentially inflating the number of ASVs detected (13). This raises concerns about the applicability of ASVs for Fungi, which could bias the overall diversity indexes and network analysis. In this study, we opted for 97% threshold OTU clustering for both bacterial and fungal communities as

reasonably accepted threshold. While a trade-off between over-splitting and lumping of species is inevitable, our approach takes into consideration potential bias derived from technical errors and DNA retrieval from these harsh environments.

Statistical analysis

Environmental parameters

Differences in environmental variables among glacier forefields were determined by performing one-way analysis of variance (ANOVA) followed by Tukey's Honestly Significant Difference (Tukey HSD) tests. Relationships between these environmental variables and locations were visualized by using a principal component analysis (PCA). Hierarchical cluster analysis was performed using Unweighted Pair Group Method with Arithmetic Mean (UPGMA) to find similarities among locations. The significance of clustering observed in the PCA was evaluated by permutational multivariate analysis of variance (PERMANOVA) (adonis; vegan package; 999 permuations (14)). Prior, the distance matrix (dist) was calculated on scaled values (scale) to standardize the environmental variables.

Enzymatic analysis

Differences in enzymatic activities across glacier forefields were determined by ANOVA followed by Tukey HSD tests. Then, the correlation between soil properties and enzymatic values were evaluated. Factors with a correlation coefficient greater than |0.7| were considered correlated; correlated factors were represented by one of them in further analysis. Relationships between microbial communities and enzymatic values were evaluated by PERMANOVA (adonis; vegan package; 999 permutations).

Microbial diversity and variation partitioning

Rarefaction curves for bacterial and fungal observed richness were generated (rarecurve; vegan package) with 500-fold step size (Figure S4). To compare the α-diversity of bacteria and fungi across locations, OTU observed richness, Simpson' diversity, Shannon diversity, Pielou' evenness, and Faith' phylogenetic diversity (Faith' PD; pd; picante package (15)) were calculated. Faith' PD requires phylogenetic distances, which were estimated based on phylogenetic trees (NJ; ape package (16)) calculated from multiple sequence alignments (alignseqs; DECIPHER package). To test the effect of locations on all α-diversity metrics, we performed an ANOVA. Pairwise differences among locations were tested using Tukey HSD. Venn diagrams were visualized with DeepVenn (17).

To visualize the variation of the microbial community compositions among glaciers, NMDS was calculated based on Bray-Curtis dissimilarities of OTU abundance matrix. Differences in microbial community compositions among locations were assessed by PERMANOVA (adonis; vegan package; 999 permuations). The environmental variables correlated to these differences were investigated in the same way. Prior to this, the microbial datasets were restricted to only those sample for which the environmental variables were measured. The final model comprised only those environmental variables with significant variance explained ($p < 0.05$) chosen based on backward selection upon significance. Variation partitioning analysis was conducted to evaluate the effects of geography and environmental variables on

the bacterial and fungal communities. First, the Principal Coordinates of Neighbour Matrices (PCNM) (pcnm; vegan package) were calculated to deconvolute total spatial variation into a discrete set of explanatory vectors on the basis of the distance matrix (distm; geosphere package) using latitudinal and longitudinal coordinates. Then, the correlation between environmental variables and distance PCNM vectors were evaluated (cor): factors with a correlation coefficient between -0.7 and 0.7 were retained (not-correlated). Finally, variation partitioning (varpart; vegan package) was calculated between the abundance OTU table of bacteria and fungi (restricted to the same sample where the environmental factors were measured), and the remaining PCNM vectors (spatial component) and environmental variables (environmental component).

Co-occurrence network analysis

We ran SPIEC-EASI with neighbourhood selection (MB), and StARS as model selection scheme.

As we expected complex interactions among bacteria and fungi, we searched for patterns across locations within networks. Thus, modules, that is clusters of nodes, were predicted based on their assortativity coefficient (cluster fast greedy (18)). We compared predicted modules from the four networks among each other to find conserved modules, i.e. modules having common microbial composition. Modules were compared by calculating Jaccard dissimilarities (vegan package) on modules' OTU compositions. Then, cluster analysis was performed on the distance matrix using the UPGMA. Furthermore, alluvial plots were created (alluvial package (19)) to allow for visual comparison of glacial modules and to inspect the importance of OTUs across glacier modules.

Random network inference were generated to test if the frequencies of observed fungal-fungal, bacterial-bacterial and bacterial-fungal associations among glacier' networks deviate from the frequencies expected by chance. Thus, based on the OTUs contained in each glacier' OTU table, we predicted a number of random associations similar to the number of associations actually inferred from this respective dataset. We repeated this process 999 times and, thus, for each random network, we determined the frequencies of the microbial associations, thereby generating a random distribution as reference distribution for each original network. In addition, we counted the number of total microbial associations shared by chance across the 999 random networks. The resulting distribution of randomly shared associations among networks was compared to the number of shared observations actually observed across glacier networks.

Community assembly

The relative influence of stochastic or deterministic processes on the microbial community assembly was calculated based on Mean Nearest Taxon Distance (MNTD) and the Nearest Taxon Index (NTI) (20, 21). Briefly, phylogenetic trees based on bacterial and fungal sequences alignment were obtained. To this end, the null MNTD was obtained by shuffling the species across the tips of the phylogeny (mntd, , "taxa.labels", 999 randomizations; picante package). Then, NTI was calculated as the negative of the difference between the observed MNTD and the null MNTD, divided by the standard deviation of the null MNTD permutations, in units of standard deviation. A mean NTI taken across all communities (plots) within a glacier

that was significantly different from the expected value of zero was interpreted as indicating an average trend towards clustering (NTI > 0) or overdispersion (NTI < 0); a two-tailed T test was used. Observed NTI values with p values < 0.05 were considered significantly structured.

The estimated contribution of assembly processes was based on Sloan Neutral Community Model (NCM) (22) and was conducted as previously described (23). First, bacterial and fungal datasets were rarefied to an equal number of OTUs corresponding to the number of reads in the smallest sample. Then, the model was calculated using the *sncm.fit* function (23) that predicts the relationship between the occurrence frequency of species in a set of local communities (i.e. individual subsamples) and their relative abundance across the global metacommunity (i.e. all four glaciers).

References

1. Thomas GW. 1996. Soil pH and soil acidity. Methods of soil analysis Part 3:475–490.

2. Schinner F, Ohlinger R, Margesin R. 1996. Methods in Soil Biology. Springer Press, Berlin.

3. Schlichting E, Blume H-P, Stahr K. 1995. Bodenkundliches Praktikum. Blackwell Wissenschaftsverlag, Berlin.

4. Bardelli T, Gómez-brandón M, Ascher-jenull J, Fornasier F, Arfaioli P, Francioli D, Egli M, Sartori G, Insam H, Pietramellara G. 2017. Effects of slope exposure on soil physico-chemical and microbiological properties along an altitudinal climosequence in the Italian Alps. Science of the Total Environment, The 575:1041–1055.

5. Fornasier F, Ascher J, Ceccherini MT, Tomat E, Pietramellara G. 2014. A simplified rapid , low-cost and versatile DNA-based assessment of soil microbial biomass. Ecological Indicators 45:75–82.

6. Caporaso JG, Lauber CL, Walters WA, Berg-Lyons D, Lozupone CA, Turnbaugh PJ, Fierer N, Knight R. 2011. Global patterns of 16S rRNA diversity at a depth of millions of sequences per sample. Proceedings of the National Academy of Sciences of the United States of America 108:4516–4522.

7. White TJ, Bruns TD, Lee SB, Taylor JW. 1990. Amplification and direct sequencing of fungal ribosomal RNA Genes for phylogenetics. PCR protocols: A Guide to Methods and Applications.

8. Martin M. 2011. Cutadapt removes adapter sequences from high-throughput sequencing reads. EMBnet.journal 17:10–12.

9. Callahan BJ, McMurdie PJ, Rosen MJ, Han AW, Johnson AJA, Holmes SP. 2016. DADA2: High-resolution sample inference from Illumina amplicon data. Nature Methods 13:581–583.

10. Wright ES. 2016. Using DECIPHER v2.0 to Analyze Big Biological Sequence Data in R. The R Journal 8:352–359.

11. Callahan B. 2018. Silva taxonomic training data formatted for DADA2 (Silva version 132) https://doi.org/10.5281/ZENODO.1172783.

12. Abarenkov K, Zirk A, Piirmann T, Pöhönen R, Ivanov F, Nilsson RH, Kõljalg U. 2021. UNITE general FASTA release for Fungi. 2020. Version 10:2021.

13. Kauserud H. 2023. ITS alchemy: On the use of ITS as a DNA marker in fungal ecology. Fungal Ecology 65:101274.

14. Oksanen J. 2015. Multivariate Analysis of Ecological Communities in R: Vegan Tutorial. 1–43.

15. Kembel SW, Wu M, Eisen JA, Green JL. 2012. Incorporating 16S Gene Copy Number Information Improves Estimates of Microbial Diversity and Abundance. PLoS Computational Biology 8:16–18.

16. Paradis E, Schliep K. 2019. ape 5.0: an environment for modern phylogenetics and evolutionary analyses in R. Bioinformatics 35:526–528.

17. Hulsen T. 2022. DeepVenn -- a web application for the creation of area-proportional Venn diagrams using the deep learning framework Tensorflow.js. arXiv https://doi.org/https://doi.org/10.48550/arXiv.2210.04597.

18. Csárdi G, Nepusz T, Müller K, Horvát S, Traag V, Zanini F, Noom D. 2023. igraph for R: R interface of the igraph library for graph theory and network analysis https://doi.org/10.5281/ZENODO.8240644.

19. Brunson J. 2020. ggalluvial: Layered Grammar for Alluvial Plots. Journal of Open Source Software 5:2017.

20. Stegen JC, Lin X, Konopka AE, Fredrickson JK. 2012. Stochastic and deterministic assembly processes in subsurface microbial communities. ISME Journal 6:1653–1664.

21. Dini-Andreote F, Stegen JC, Van Elsas JD, Salles JF. 2015. Disentangling mechanisms that mediate the balance between stochastic and deterministic processes in microbial succession. Proceedings of the National Academy of Sciences of the United States of America 112:E1326–E1332.

22. Sloan WT, Lunn M, Woodcock S, Head IM, Nee S, Curtis TP. 2006. Quantifying the roles of immigration and chance in shaping prokaryote community structure. Environmental Microbiology 8:732–740.

23. Burns AR, Stephens WZ, Stagaman K, Wong S, Rawls JF, Guillemin K, Bohannan BJM. 2016. Contribution of neutral processes to the assembly of gut microbial communities in the zebrafish over host development. ISME Journal 10:655–664.
